# Association between CLOCK gene polymorphisms with circadian rhythm, chrononutrition, dietary intake, and metabolic parameters in adolescents

**DOI:** 10.3389/fpubh.2024.1435460

**Published:** 2024-12-18

**Authors:** Gizem Özata Uyar, Hilal Yıldıran, Demet Teker-Düztaş, Buket Dalgic, Nazmi Mutlu Karakaş, Mahmut Orhun Çamurdan, Filiz Başak Ergin, Fatih Suheyl Ezgü

**Affiliations:** ^1^Department of Nutrition and Dietetics, Kırıkkale University, Kırıkkale, Türkiye; ^2^Department of Nutrition and Dietetics, Gazi University, Ankara, Türkiye; ^3^Department of Pediatric Gastroenterology and Hepatology, Gazi University, Ankara, Türkiye; ^4^Department of Pediatrics, Gazi University, Ankara, Türkiye; ^5^Department of Pediatric Endocrinology, Faculty of Medicine, Gazi University, Ankara, Türkiye; ^6^Department of Pediatric Metabolism and Nutrition, Gazi University, Ankara, Türkiye

**Keywords:** CLOCK gene, chrononutrition, adolescents, obesity, circadian rhythm

## Abstract

**Introduction:**

Circadian Locomotor Output Cycles Kaput (CLOCK) is one of the transcription factors from the positive end of the molecular clock and regulates biological rhythm in mammals. Studies have shown that genetic variations in the CLOCK genes are associated with chronotype, sleep patterns, obesity, dietary energy, and nutrient consumption. Although interest in the field of chrononutrition continues to increase, investigations into the temporal aspects of dietary habits in adolescents are notably limited. This study aims to investigate the relationship between CLOCK rs3749474, rs4580704, and rs1801260 polymorphisms and circadian rhythm, metabolic parameters along with chrononutrition in adolescents.

**Materials and methods:**

This cross-sectional study included a total of 300 adolescents between the ages of 11 and 18. Anthropometric measurements, dietary intake, chrononutrition, circadian as well as biochemical and lifestyle data were collected. CLOCK rs3749474, rs4580704, and rs1801260 gene polymorphisms were analyzed.

**Results:**

The CLOCK rs3749474 minor T allele carriers showed a statistically significant increased risk of being overweight or obese (OR: 2.106, *p* = 0.003). The minor G allele carriers of the CLOCK rs4580704 indicated statistically increased dietary energy intake and eating jetlag (*p* < 0.05). The frequency of snacking after the last meal was positively correlated with body mass index (BMI) z-scores in minor allele carriers of the CLOCK rs3749474 (β = 0.134, *p* = 0.003) and rs4580704 (β = 0.142, *p* = 0.012) variants. The minor G allele carriers of CLOCK rs4580704 revealed a negative link between breakfast frequency along with BMI z-scores (β = −0.178, *p* = 0.009). Individuals with the rs1801260 minor G allele showed a positive link between BMI z score and meal frequency (β = 0.367, *p* = 0.049). In CLOCK gene variants, minor allele carriers in addition to non-carriers had similar biochemical parameters and distribution of dietary intake at meal (*p*> 0.05).

**Conclusion:**

These results suggest that the impact of some chrononutrition behaviors on BMI z-scores are partially modulated by the variability in the CLOCK gene variants. Chrononutrition may be important in the shift toward “personalized nutrition” based on gene-diet interactions. For this reason, new dietary approaches may be implemented, including the circadian distribution of macronutrients and chrononutrition behaviors according to genotype. However, studies with larger samples in different populations are needed.

## 1 Introduction

The dramatic increase in the prevalence of overweight and obesity among children as well as adolescents in recent years has become a significant public health concern, with an epidemic level today (1, 2). According to the Centers for Disease Control and Prevention (CDC) data, the prevalence of obesity among children along with adolescents aged 2 to 19 reached 19.7% between 2017 and 2020, affecting 14.7 million individuals (3).

Adolescence is a crucial stage during which many behavioral patterns, such as eating habits and physical activity, begin developing (4). During this time, the existence of excess weight along with obesity leads to a decline in cardio-metabolic health and a rise in cardiovascular mortality as well as a disease burden during adulthood (5, 6). Recently, it has been indicated that circadian rhythm disorders resulting from behavioral and lifestyle choices cause an increase in the risk of obesity and chronic diseases (7). For this reason, regulating the circadian rhythm may be effective in addition to the dietary and physical activity interventions recommended for treating obesity (8). In mammals, daily cycles of feeding/fasting, sleep/wake, along with body temperature, which involve multiple neuroendocrine pathways, influence the circadian rhythm by regulating energy intake and energy expenditure (9–11). Circadian rhythm disorders disrupt the integration of metabolic systems resulting in obesity, inflammation, metabolic-cardiovascular problems, diabetes, insulin resistance, cancer, and intestinal microbiota disorders (7, 9, 12–14).

Beyond the significance of nutrient type and quantity, recent studies in circadian science have shifted attention toward the influence of meal timing on health outcomes (15–17). Consequently, the chrononutrition term, which refers to the timing of food intake within the circadian rhythm, has emerged (18–21). The concept of chrononutrition encompasses many components, such as meal regularity, meal frequency, meal time, skipping breakfast, the largest meal, dinner meal, evening delay, night eating, eating window, as well as food consumption at meals (22–24). Studies have indicated that factors such as delay in meal times, skipping breakfast in addition to consuming snacks at night increase the risk of being overweight/obese (15, 16), and limiting the eating window (e.g., 10 h per 24 h per day) may help weight loss success and improve metabolic health (17). A positive relationship was also found between eating jetlag, which is an indicator of the variability of meal timing on weekends and weekdays, as well as BMI (25).

Circadian clocks are molecular machines that regulate daily timing, coordinating a variety of molecular, physiological, and behavioral events (26). The four primary proteins comprise the molecular machinery of the circadian rhythm and each controls the expression of other genes downstream in a negative feedback loop. The heterodimeric connection between two transcription factors— Circadian Locomotor Output Cycles Kaput (CLOCK) and The Brain and Muscle ARNT-like 1 (ARNTL, also known as BMAL1)—plays a key role in the control of this loop (27, 28). In particular, transcription of BMAL1 and CLOCK genes, which are primarily expressed in the forebrain, leads to the heterodimerization in the cytoplasm of the BMAL1:CLOCK complex, which translocates into the nucleus and binds to Enhancer Box (E-Box), and rhythmically induce the transcription of other circadian clock genes (29). Period (PER) and cryptochrome (CRY) genes comprise the negative feedback loop. Among the several target genes, BMAL1 and CLOCK also stimulate the expression of the genes that make up the negative loop of the molecular clock, including CRY and PER (30). In the cytoplasm, PER and CRY bind to produce a complex that transfers into the nucleus. They inhibit the transcriptional activity of the BMAL1:CLOCK complex after their translation and nuclear accumulation (29, 30). These central clock components modulate hundreds of other clock-controlled genes (CCGs) (31). Thus, mutations in clock-related genes alter transcriptional regulators, which affect hypothalamic feeding behavior and peripheral energy metabolism, causing several metabolic disorders (32, 33) such as hypercholesterolemia, hyperglycemia, sleep disturbances, metabolic disorders, and cardiovascular diseases (34).

Circadian Locomotor Output Cycles Kaput (CLOCK) is a transcription factor from the molecular clock. This transcription factor also influences energy metabolism by impacting several metabolic pathways, such as glucose and lipid metabolism, in specific organs including muscle, liver, and adipose tissue (35). In addition, the CLOCK gene is located on chromosome 4q12 (36). This region was associated with obesity (37). In recent years, the relationship between CLOCK gene variants and obesity along with the underlying mechanisms have attracted attention (38–40). In particular, studies have focused on the rs3749474, rs4580704, and rs1801260 genetic variants of the CLOCK gene (8, 39, 41, 42). Researchers have found that differences in the CLOCK rs3749474, rs4580704, and rs1801260 gene variants are associated with chronotype, sleep patterns, obesity, dietary energy, and nutrient consumption (8, 39, 41, 42). On the other hand, some genetic variations could be responsible for individual responses to various diets (43). While BMAL1 is an essential partner in the circadian clock system (29), we chose to focus on the CLOCK gene because its impact on energy metabolism, obesity, and eating behaviors is more widely studied. Although interest in chrononutrition continues to increase, most studies in the literature have been conducted in adults, and investigations into the temporal aspects of dietary habits in adolescents are notably limited. Furthermore, there is a lack of research in the existing literature that comprehensively investigates the effect of the interplay between SNPs (Single Nucleotide Polymorphisms) in the CLOCK gene, chrononutrition behaviors along with energy distribution in meals on Homeostatik Model Assessment for Insulin Resistance (HOMA-IR) and BMI z-scores. Therefore, the primary aim of this study is to determine the frequencies of CLOCK rs3749474, rs4580704 as well as rs1801260 gene polymorphisms in Turkish adolescents and to detect differences in chronotype, chrononutrition, sleep quality, anthropometric measurements, and a variety of biochemical parameters according to genotypes. The second aim of this study is to examine how CLOCK gene polymorphisms interact with chronotype and sleep quality to affect dietary intake. The third goal is to evaluate the effect of CLOCK gene polymorphisms' interacting with chrononutrition, sleep quality, and chronotype score on BMI z-scores.

Considering all these parameters, this study will shed important light on the existing literature. For this reason, this study intends to contribute to the evolving landscape of circadian biology by systematically evaluating the intricate relationships between clock gene polymorphism, chronotype, and chrononutrition in adolescents.

## 2 Methods

### 2.1 Participants and study design

**Figure 1:**
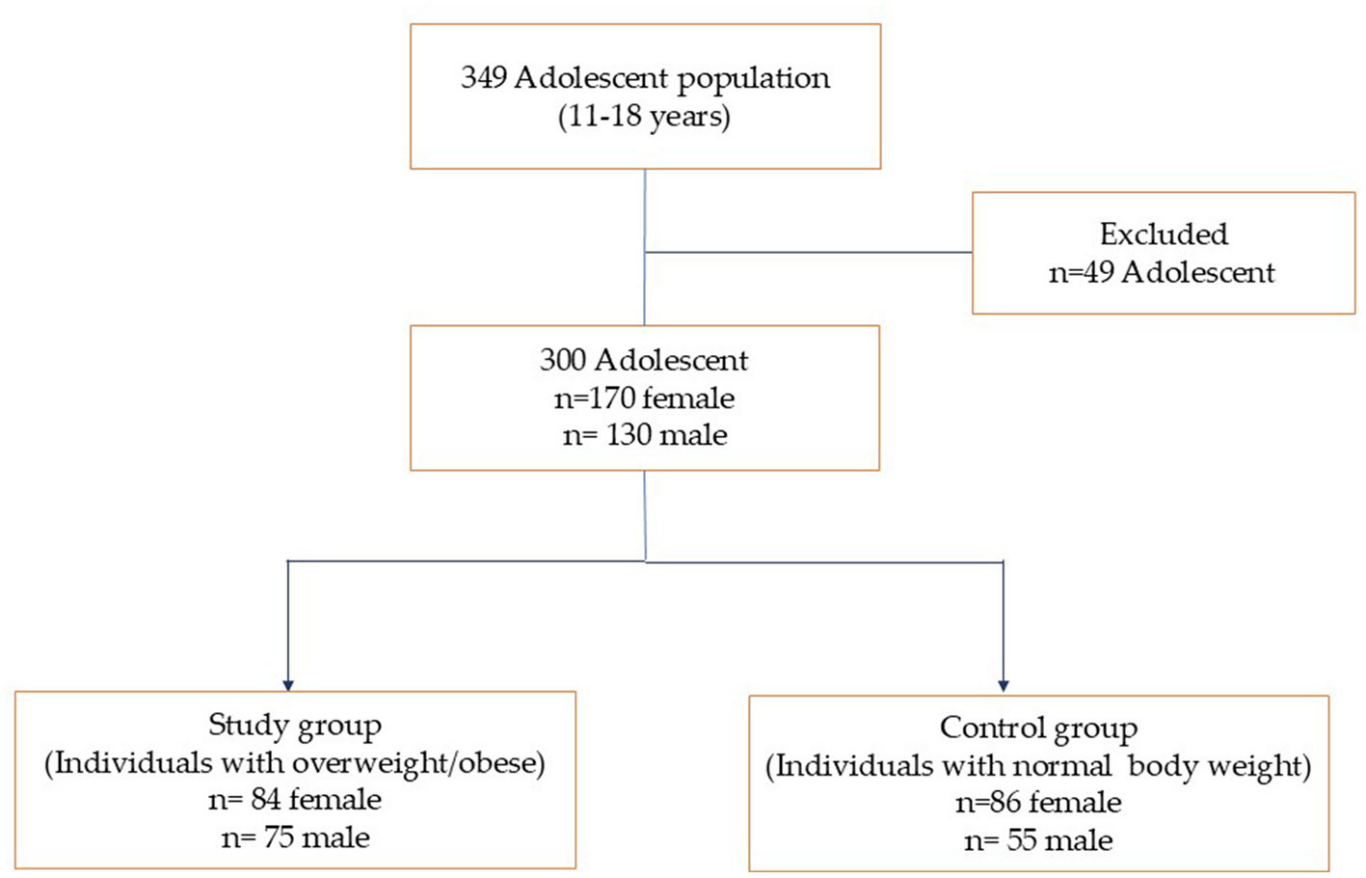
Study flow diagram.

This cross-sectional study was conducted at the Gazi University Department of Pediatric Gastroenterology, the Department of Pediatric Endocrinology, and the General Outpatient clinic from August 2020 to September 2021. A total of 300 adolescents between the ages of 11 and 18 were included in the study, comprising 159 individuals with overweight or obese (Study group → male: 75, female: 84) and 141 individuals with normal body weight (Control group → male: 55, female: 86) (Figure 1). Participants were recruited from individuals who applied to the outpatient clinic as of the specified date, met the inclusion criteria, and were referred by the attending doctor. Those who agreed to participate were included in the study.

Individuals were chosen based on the following inclusion criteria: (1) age range of 11–18 years, (2) according to BMI z-scores, being overweight, obese, or normal weight. The following conditions were excluded: Having relatives or people of different ethnic origins (to minimize genetic variability that could confound the results, as genetic polymorphisms can vary significantly across ethnic groups, potentially affecting the accuracy and relevance of the findings in Turkish adolescents), a history of a syndromic or endocrinological disease that causes obesity, thyroid dysfunction (hyperthyroid and hypothyroid), Type 1 or Type 2 diabetes, cardiovascular diseases, kidney diseases, autoimmune or metabolic liver diseases, major psychiatric disorder, taking medication that affects sleep patterns and following a special diet were also taken out of consideration. However, 49 adolescents were excluded from the study due to missing data, fear of donating blood, lack of biochemical data, or failure to provide dietary intake records for two days.

All participants along with their parents provided written informed consent for this study, which was conducted in compliance with the Declaration of Helsinki's standards. The Ethics Committee of Clinical Research at Gazi University approved the protocol research (No: 370, 08.06.2020).

### 2.2 Anthropometric measurements

Analysis on the body weight and composition of adolescents were measured with a Tanita BC 545N body analyzer (bioelectric impedance device-BIA) (44). Height was measured with a stadiometer. BMI was calculated using the following formula: “BMI = [body weight (kg)/(height [m])^2^]”. The WHO AnthroPlus program was used to evaluate the BMI z-scores of individuals. Age- and sex-specific standardized BMI scores (BMI z-scores) were calculated based on the growth chart developed by WHO (45). Waist circumference was measured using a non-stretch tape measure, taking into account standard methods (46). The research dietitian performed all anthropometric measures and surveys.

### 2.3 Physical activity level

Physical activity levels (PAL) were determined through the 24-h physical activity record (47, 48). According to the PAL classification obtained, since most individuals are very lightly active and sedentary individuals, the lightly active group was combined with the sedentary group. The medium active group was added to the heavy active group.

### 2.4 Dietary intake

Dietary intake was determined during 2 days, one weekday and one weekend, using the 24-h dietary recall method conducted by a trained research dietitian. The meal times of the individuals were also recorded. The “Food and Nutrition Photo Catalogue” was used to record food and portion amounts accurately (49). The dietary intake of participants was analyzed using Nutrition Information Systems (Beslenme Bilgi Sistemi-BeBiS 9.0), which complies with Turkish food regulations (50).

After reviewing the literature and considering meal definitions, it was determined that meals should be classified based on Turkish cultural patterns. However, there is no standard definition for the classification of meals. For breakfast meals, the interval between 05:00 and 10:00 on weekdays and between 05:00 and 11:00 on weekends is accepted (40). The lunchtime frame is 12:00–17:00, while dinner is 17:01–20:00 (51–56). Consumption of energy after 20:00 was defined as night (57).

### 2.5 Chrononutrition

A review of previously published study questions on chrononutrition was made (22, 58, 59). The average estimates of sleep and eating timing for weekdays and weekends, which included bedtime, the first eating event, and the last eating event, were used to calculate key chrononutrition variables such as evening latency, evening eating, and eating window. Additionally, we assessed the weekly frequencies of breakfast skipping, largest meal consumption, meal timings, snacking after the last meal, and night eating to evaluate chrononutrition behavior (22).

The eating window was calculated as the time between the first and last energy-containing eating events of the day (22, 60). The average weekly eating window was calculated as follows: [5 x weekday eating window (h) + 2 x weekend eating window (h)]/7.

The eating midpoint was calculated as the mid between the first and last meal timing. It is calculated similarly to the recommended method for predicting midpoint sleep. The eating midpoint was calculated as follows: Eating midpoint (local time) = ([Timing of the last meal − Timing of the first meal]/2) + Timing of the first meal (60). The average weekly eating midpoint was calculated as follows: [5 x weekday eating midpoint (h) + 2 x weekend eating midpoint (h)]/7.

Eating jet lag was calculated in hours as follows: Eating midpoint on weekends-Eating midpoint on weekdays (60).

Morning latency (minutes) is the time interval between when a person wakes up and their first eating event, whereas evening latency (minutes) is the time span from the last eating event to the onset of sleep (22). The average morning and evening latencies were calculated as follows: [5 x weekday morning/evening latency + 2 x weekend morning/evening latency]/7.

### 2.6 Pittsburgh sleep quality index

Buysse and colleagues developed the Pittsburgh Sleep Quality Index (PSQI) in 1989 to assess the sleep quality of patients over the last month (61). The Turkish validity and reliability of the scale were confirmed by Agargün (62). It has 24 questions, 19 of which are scored. PSQI is calculated by scoring each component 0–3 points. Total scores range from 0 to 21, and >5 indicates poor sleep quality (62).

### 2.7 Chronotype and circadian parameters

The Childhood Chronotype Questionnaire was used to determine chronotype. It was developed by Werner et al. (63). The Turkish validity and reliability study was conducted by Dursun et al. (64). The questionnaire consists of 27 questions in a mixed format, filled out by the family, and questions in many areas (64). School days along with holidays/free days are evaluated separately; the total score varies between 10 and 48. According to the total scores, ≤ 23 scores are classified as “morning chronotype,” 24–32 scores are classified as “intermediate chronotype,” and ≥33 scores are classified as “evening chronotype.”

Adolescents were questioned about weekday/school day and weekend/holiday sleep and wake-up timings. The weekly sleep duration was [5 × weekday sleep (h) + 2 × weekend sleep (h)]/7 (65). The absolute difference in workday and weekend sleep midpoints is calculated in hours to determine social jetlag (66).

### 2.8 Biochemical parameters

After at least 8 h of fasting, venous blood samples were taken by experienced nurses in the blood collection department of Gazi University Hospital. Venous blood samples were taken by nurses in the morning after fasting for at least 8 h. Serum fasting glucose, insulin, total cholesterol, high-density lipoprotein (HDL) cholesterol, low-density lipoprotein (LDL) cholesterol, triglycerides, alanine aminotransferase (ALT), and aspartate aminotransferase (AST) were assayed in Gazi University Hospital Medical Biochemistry Laboratory according to conventional laboratory standard methods. HOMA-IR was obtained with the formula fasting glucose (mg/dL) x fasting insulin (IU ml)/405 (67).

### 2.9 Gene selection and genotyping

The CLOCK rs180260, rs4580704, and rs3749474 gene variants were selected in light of research conducted on various populations in the literature. Because these variants were linked to metabolic parameters, food consumption, sleep quality, obesity, and chronotype (38, 42, 68–70). In this study, genotype frequencies of CLOCK SNPs, rs3749474, rs4580704, and rs180260 in Turkish adolescents provide Hardy-Weinberg equilibrium (*p* = 0.898, *p* = 0.649, *p* = 0.927, respectively).

For genetic analysis, 3 mL of blood sample was taken into an Ethylenediamine tetraacetic acid (EDTA) tube (BD Vacutainer^®^ EDTA Tubes, 367863/USA). DNA was isolated from blood samples using routine DNA isolation sets (Diagen Biotechnology, BLD-5295/Turkey). Nucleic acid loads of the samples obtained from the total DNA extraction process were measured with the Colibri Microvolume Spectrometer (Titertek-Berthold, Germany). To normalize the DNA amounts across all samples, the concentration was adjusted to an average of 100 ng/μL. This step ensured that equal amounts of DNA were used for each sample, improving the reliability of the analysis results. Additionally, the DNA quality and integrity were verified using a Nanodrop spectrophotometer and agarose gel electrophoresis before the experiment, ensuring consistency between samples.

SNP analysis was performed according to the Taqman probe principle using real time PCR technology. Primer probes required for the identification of 3 selected SNPs specific to the CLOCK gene were ordered using the TaqMan™ SNP Genotyping Assay (ThermoFisher, https://www.thermofisher.com/taqman/snp/assay/query) database, which was prepared based on previous studies according to the Taqman principle. CLOCK gene regions (rs1801260, rs4580704, and rs3749474) were performed through Real time Polymerase Chain Reaction (PCR) using ThermoFisher TaqMan™ SNP Genotyping Assay kits (Assay ID: C___8885584_20, C__28028791_10, C__26405955_10) according to the manufacture protocol (Thermo Fisher, Carlsbad, CA). Real time PCR analysis was performed on the ABI 7500 FAST (Thermo Fisher, USA) device.

SensiFAST™ Probe No-ROX Kit (Bioline, UK, BIO-86020) was used for real-time PCR reactions. SensiFAST Probe No-ROX 2x Mix, TaqMan SNP Genotyping Assays 20x Primer Mix, and 4.5 μl DNA were used for 10 μl reaction mixture (71). The reaction was carried out using ABI 7500 FAST 8-strip tubes, with initial denaturation at 95°C for 10 min, 40 repetitions at 95°C for 15 s, and fragment amplification at 61°C for 60 s (reading).

Fluorescence dye-labeled probes were utilized to identify mutations on the CLOCK gene region for the T (FAM) and C (VIC) alleles of rs3749474, the G (FAM) along with C (VIC) alleles of rs4580704, in addition the G (FAM) and A (VIC) alleles of rs1801260.

Genotypes according to these radiations in the real time PCR device results are processed in the table as follows. CLOCK rs3749474; CC (normal), TT (mutant), TC (heterozygous); CLOCK rs4580704; CC (normal), GG (Mutant), CG (Heterozygous); CLOCK rs1801260; AA (normal), GG (mutant) and AG (heterozygous).

### 2.10 Statistical analysis

The data obtained from the study were analyzed using the IBM Statistical Package for the Social Sciences (SPSS) version 24.0 statistical package program (Chicago, IL, USA). The suitability of the variables to normal distribution was determined by visual (histogram and probability graphs) in addition to analytical methods (Shapiro-Wilk tests). Nonnormally distributed data were transformed using the natural log (Ln) value where relevant. The categorical data were represented as frequencies and percentages, while the quantitative data were given as mean± standard deviation (SD). A dominant model was used, in which common homozygous genotypes (non-minor allele carriers) were compared to combined variant homozygous (two minor allele carriers), and heterozygous genotypes (one minor allele carriers), due to the limited number of people with variant homozygous genotypes. Pearson's x^2^ test or Fisher's exact test was used to test frequency differences. Pearson's x^2^ test was used to test genotype frequencies in subjects for Hardy Weinberg equilibrium (HWE). Independent Samples t-test was used to compare two independent groups for crude means. Multivariate adjustments of the associations were also performed using an analysis of covariance. The results were adjusted for potentially confounding factors such as age, gender, BMI z-score, PAL levels, and energy intake wherever needed.

Binary logistic regression was conducted to assess the risk of overweight/obesity based on the genotypes of individuals. The odds ratio (OR) and its 95% confidence intervals (CI) were calculated after adjusting for age and gender.

General linear model was used to evaluate the effect of CLOCK rs3749474, rs4580704, and rs1801260 gene variants' interactions with chronotype and sleep quality on dietary intake. The models were adjusted for age, sex, BMI z-scores, and total energy intake. In the models where total energy intake was a dependent variable, total energy was not included as a covariate. Other *p* values were obtained from linear regression analysis for each group.

Multivariable linear regression analysis was used to assess the effect of interaction of CLOCK gene variants with chrononutrition, sleep quality, and chronotype on BMI z-score. Confounding factors such as age, gender, as well as physical activity level for BMI z-score were adjusted. A model was created by calculating the weekly averages of morning latency, evening latency, eating window, and eating midpoint values, separately for weekdays and weekends, based on chrononutrition behaviors. Linear regression results were reported as (β) coefficients and confidence intervals (95% CI). Logistic regression analyzes were used to identify meal energy distribution and interactions of CLOCK gene variants that could influence overweight/obesity and the evening chronotype. Odds ratios (ORs) and confidence intervals (95% CI) were calculated after adjusting for age, gender, physical activity level, and BMI z-score. Statistical significance was accepted as p < 0.05.

## 3 Results

Among adolescents with overweight or obese (study group), 47.2% were male (*n* = 75) and 52.8% were female (*n* = 84), while among those with normal weight (control group), 39.0% were boys (*n* = 55) and 61.0% were girls (*n* = 86) (*p* = 0.154). The mean age and the standard deviation of individuals in the study and control groups were 14.0 ± 2.18 years and 15.0 ± 1.76 years, respectively, and the difference between the groups for age was found to be statistically significant (*p* < 0.001) (Data not shown in table).

### 3.1 Genotype distributions of individuals and anthropometric measurements, physical activity levels, and biochemical parameters

CLOCK rs3749474 gene in comparison to CC genotype: Adolescents with the CT genotype showed a significantly elevated risk of being overweight or obese, with an odds ratio (OR) of 2.134 (*p* = 0.004) when compared to those with the CC genotype. In contrast, the TT genotype was associated with a slightly lower increased risk (OR = 2.013) compared to CC; however, this difference was not statistically significant. Individuals carrying the minor risk allele (CT+TT genotypes combined) had a 2.106-fold increased risk of being overweight or obese compared to those with the CC genotype (*p* = 0.003). This finding underscores a significant association between the CLOCK rs3749474 minor allele and elevated overweight/obesity risk among adolescents. For the CLOCK rs4580704 and rs1801260 genotypes, no significant differences were observed in the distribution between overweight/obese and normal-weight groups, suggesting no apparent association with BMI status for these variants (Table 1).

**Table 1:** Genotype frequencies of CLOCK rs3749474, rs4580704, and rs1801260 among individuals with normal weight and overweight/obese.

**CLOCK SNPs**	**Total (*n* = 300)**	**Normal (*n* = 141)**	**Overweight/obese (*n* = 159)**	**OR (%95CI)**	**p^a^**
**rs3749474**				
**Codominant**				
CC	112 (37.3)	66 (46.8)	46 (28.9)	Reference	
CT	146 (48.7)	57 (40.4)	89 (56.0)	2.134 (1.269–3.588)	**0**.**004**
TT	42 (14.0)	18 (12.8)	24 (15.1)	2.013 (0.953–4.250)	0.067
**Dominant**				
CC	112 (37.3)	66 (46.8)	46 (28.9)	Reference	
CT+TT	188 (62.7)	75 (53.2)	113 (71.1)	2.106 (1.286–3.449)	**0**.**003**
**rs4580704**				
**Codominant**				
CC	130 (43.3)	61 (43.3)	69 (43.4)	Reference	
CG	129 (43.0)	53 (37.6)	76 (47.8)	1.147 (0.685–1.921)	0.602
GG	41 (13.7)	27 (19.1)	14 (8.8)	0.468 (0.219–1.001)	0.050
**Dominant**				
CC	130 (43.3)	61 (43.3)	69 (43.4)	Reference	
CG+GG	170 (56.7)	56.7 (26.7)	90 (56.6)	0.922 (0.570–1.491)	0.740
**rs1801260**				
**Codominant**				
AA	166 (55.3)	75 (53.2)	91 (57.2)	Reference	
AG	113 (37.7)	54 (38.3)	59 (37.1)	0.873 (0.530–1.438)	0.593
GG	21 (7.0)	12 (8.5)	9 (5.7)	0.678 (0.263–1.748)	0.421
**Dominant**				
AA	166 (47.0)	75 (53.2)	91 (57.2)	Reference	
AG+GG	159 (53.0)	66 (46.8)	68 (42.8)	0.839 (0.521–1.350)	0.469

^a^Age and gender were adjusted. Logistic regression analysis. OR, Odds ratio; CI, Coefficient intervals. The statistically significant p values are shown in bold (p < 0.05).

The age, some anthropometric measurements, body composition, biochemical parameters, and physical activity levels of the individuals according to the genotypes are given in Table 2. After adjusting for age and gender, individuals carrying the minor allele (CT+TT) of the CLOCK rs3749474 gene exhibited significantly higher BMI z-scores, body fat percentages in addition to trunk fat percentages than those with the CC genotype (non-minor allele carriers). These findings suggest a potential association between the rs3749474 minor allele and increased adiposity. In contrast, the CLOCK rs4580705 (except for height) and rs1801260 genotypes did not show significant differences in anthropometric measurements between minor allele carriers and non-carriers (*p* > 0.05). For all three CLOCK SNPs (rs3749474, rs4580705, and rs1801260), biochemical parameters including fasting blood glucose, insulin levels, HOMA-IR, and lipid profiles did not differ significantly between minor allele carriers and non-carriers (*p* > 0.05). Notably, AST levels were significantly higher in CLOCK rs4580704 G minor allele carriers compared to non-minor allele carriers (*p* = 0.014). However, after adjusting for age, gender, and BMI z-score, this difference lost statistical significance (*p* = 0.096). Regarding physical activity, individuals with the common homozygous AA genotype of CLOCK rs1801260 were significantly more active, with an average activity level of 1.6 ± 0.29, compared to 1.4 ± 0.26 in minor allele carriers (*p* = 0.023). This finding suggests a potential association between the AA genotype and higher physical activity levels (*p* = 0.023).

**Table 2:** Age, some anthropometric measurements, body composition, biochemical parameters, and physical activity levels of the adolescents according to the CLOCK genotypes.

	**CLOCK rs3749474**				**CLOCK rs4580704**				**CLOCK rs1801260**			
	**CC (*****n*** = **112)**	**CT**+**TT (*****n*** = **188)**	*p* ^a^	*p* ^b^	**CC (*****n*** = **130)**	**CG** + **GG (*****n*** = **170)**	*p* ^a^	*p* ^b^	**AA (*****n*** = **166)**	**AG**+**GG (*****n*** = **134)**	*p* ^a^	*p* ^b^
Age^β^	14.7 ± 2.07	14.4 ± 2.06	0.223	-	14.5 ± 2.11	14.4 ± 2.03	0.546	-	14.5 ± 2.09	14.4 ± 2.02	0.775	-
**Anthropometric measurements** ^β^												
Body weight (kg)	65.9 ± 19.29	67.7 ± 17.45	0.416	0.236	65.3 ± 15.70	68.3 ± 19.73	0.142	0.272	67.6 ± 18.09	66.2 ± 18.25	0.502	0.786
Height (cm)	163.9 ± 9.91	162.0 ± 9.16	0.102	0.207	161.4 ± 8.68	163.7 ± 9.95	**0**.**039**	**0**.**037**	163.4 ± 9.81	161.9 ± 9.00	0.176	0.362
BMI z-scores	1.1 ± 1.49	1.5 ± 1.38	**0**.**017**	**0**.**029**	1.3 ± 1.36	1.4 ± 1.49	0.717	0.871	1.4 ± 1.42	1.3 ± 1.46	0.720	0.803
Body fat (%)	27.6 ± 9.94	30.4 ± 8.98	**0**.**015**	**0**.**017**	29.4 ± 8.74	29.3 ± 9.95	0.931	0.759	29.4 ± 9.54	29.3 ± 9.32	0.913	0.642
Trunk fat (%)	23.2 ± 9.98	26.0 ± 9.92	**0**.**018**	**0**.**021**	24.8 ± 9.65	25.1 ± 10.32	0.824	0.688	25.2 ± 10.26	24.7 ± 9.76	0.687	0.575
Waist circumference (cm)	81.9 ± 15.31	84.5 ± 15.11	0.161	0.158	82.0 ± 14.00	84.7 ± 16.02	0.125	0.395	84.1 ± 15.21	82.8 ± 15.24	0.453	0.734
Waist/height	0.50 ± 0.09	0.52 ± 0.09	**0**.**048**	0.076	0.50 ± 0.09	0.52 ± 0.09	0.443	0.854	0.52 ± 0.09	0.51 ± 0.09	0.739	0.915
**Biochemical parameters**												
Fasting glucose (mg/dL)	84.1 ± 12.28	85.4 ± 11.02	0.319	0.868	84.8 ± 11.11	85.0 ± 11.83	0.973	0.879	85.8 ± 11.13	83.8 ± 11.89	0.125	0.106
Insulin (I/U)	12.5 ± 9.99	14.1 ± 10.72	0.087	0.777	14.2 ± 12.93	13.0 ± 8.09	0.801	0.910	13.4 ± 10.89	13.6 ± 9.95	0.553	0.492
HOMA-IR	2.7 ± 2.63	3.1 ± 2.57	0.082	0.767	3.2 ± 3.30	2.8 ± 1.88	0.816	0.890	2.9 ± 2.60	167.1 ± 28.15	0.829	0.802
Total cholesterol (mg/dL)	165.6 ± 25.14	166.3 ± 31.46	0.823	0.963	166.3 ± 31.90	165.9 ± 27.09	0.922	0.960	167.1 ± 28.15	164.7 ± 30.54	0.483	0.441
LDL cholesterol (mg/dL)	98.2 ± 19.59	98.7 ± 26.82	0.864	0.916	98.9 ± 27.66	98.2 ± 21.53	0.827	0.803	98.9 ± 23.09	98.1 ± 25.88	0.796	0.807
HDL cholesterol (mg/dL)	48.3 ± 10.15	48.2 ± 9.82	0.896	0.408	49.0 ± 9.88	47.6 ± 9.96	0.227	0.461	48.6 ± 10.00	47.7 ± 9.86	0.421	0.174
Triglycerides (mg/dL)	101.4 ± 61.86	99.1 ± 48.69	0.741	0.180	95.5 ± 49.3	103.4 ± 57.1	0.176	0.161	101.1 ± 58.17	98.6 ± 48.25	0.993	0.964
VLDL (mg/dL)	20.3 ± 12.36	19.9 ± 9.79	0.799	0.202	19.1 ± 9.85	20.8 ± 11.46	0.159	0.142	20.34 ± 11.68	19.7 ± 9.65	0.914	0.950
Non-HDL (mg/dL)	116.7 ± 26.4	116.8 ± 32.08	0.973	0.381	115.4 ± 33.43	117.9 ± 27.24	0.447	0.495	118.0 ± 28.61	115.3 ± 31.79	0.546	0.599
ALT (I/U)	19.7 ± 19.33	23.3 ± 22.4	0.052	0.512	20.7 ± 17.13	23.0 ± 24.09	0.587	0.826	22.5 ± 21.71	21.3 ± 20.95	0.520	0.849
AST (I/U)	24.2 ± 8.96	24.8 ± 10.23	0.606	0.948	23.2 ± 8.51	25.6 ± 10.53	**0**.**014**	0.096	24.8 ± 9.50	24.3 ± 10.12	0.460	0.764
**Physical activity level** ^**β**^	1.5 ± 0.27	1.5 ± 0.29	0.421	0.163	1.5 ± 0.27	1.5 ± 0.29	0.492	0.806	1.6 ± 0.29	1.4 ± 0.26	**0**.**018**	**0**.**023**
Sedentary/low	92 (82.1)	139 (73.9)	0.102		104 (80.0)	127 (74.7)	0.280		117 (70.5)	114 (85.1)	**0.003**	
Moderate/high	20 (17.9)	49 (26.1)			26 (20.0)	43 (25.3)			49 (29.5)	20 (14.9)		

The association of the CLOCK gene variants with anthropometric measurements, biochemical parameters, and physical activity was determined by linear regression. Pearson's Chi-square test was used for categorical variables. p^a^ values were unadjusted (Independent t test to analyze differences between minor allele carrier and non-carrier groups). p^b^ values were adjusted for age and gender for anthropometric measurements. p^b^ values were adjusted for age, gender, and BMI z-score for biochemical parameters and PAL levels. ^β^Adjusting variables not included in the model when examined as dependent variables and categorical comparison. The statistically significant p values are shown in bold (p < 0.05). BMI, body mass index; HOMA-IR, Homeostatik Model Insülin Resistance; LDL, low density lipoprotein; HDL, high density lipoprotein; VLDL, very low density lipoprotein; ALT, alanine transaminase; AST, aspartate aminotransferase.

### 3.2 Circadian and chrononutrition parameters

Table 3 presents circadian parameters and chrononutrition behaviors among adolescents, categorized by CLOCK genotypes. CLOCK rs3749474, rs4580704 and rs1801260 variants showed no significant difference in terms of sleep quality, chronotype score, and some circadian parameters (Sleep onset time, wake-up time, sleep duration) according to genotypes (*p* > 0.05). However, notable differences emerged in chrononutrition habits based on genotype. Adolescents with the CLOCK rs3749474 minor T allele reported later lunch times (*p* = 0.017) and longer morning latency (the delay between waking and first eating) on weekends (*p* = 0.048) compared to those without the T allele. In contrast, individuals carrying the CLOCK rs1801260 minor allele tended to eat lunch earlier than non-carriers (*p* = 0.004). Furthermore, eating jetlag duration—the discrepancy in eating times between weekdays and weekends—differed significantly by genotype for the CLOCK rs4580704 variant. Individuals with the CC genotype had a shorter eating jetlag (67.7 ± 56.64 min) compared to those with the minor G allele (81.0 ± 61.28 min, *p* = 0.034).

**Table 3:** Circadian parameters and chrononutrition behaviors of adolescents according to CLOCK genotypes.

	**CLOCK rs3749474**				**CLOCK rs4580704**				**CLOCK rs1801260**			
	**CC (*****n*** = **112)**	**CT**+**TT (*****n*** = **188)**	*p* ^a^	*p* ^b^	**CC (*****n*** = **130)**	**CG**+**GG (*****n*** = **170)**	*p* ^a^	*p* ^b^	**AA (n** = **166)**	**AG**+**GG (*****n*** = **134)**	*p* ^a^	*p* ^b^
PSQI (score)	5.2 ± 3.51	5.5 ± 3.19	0.341	0.210	5.6 ± 3.14	5.3 ± 3.45	0.465	0.521	5.3 ± 3.16	5.6 ± 3.50	0.388	0.897
Good (< 5)	69 (61.6)	112 (59.6)	0.728	-	78 (60.0)	103 (60.6)	0.918	-	102 (61.4)	79 (59.0)	0.661	-
Bad (≥5)	43 (38.4)	76 (40.4)			52 (40.0)	67 (39.4)			64 (38.6)	55 (41.0)		
Chronotype (score)	31.9 ± 6.09	32.1 ± 5.95	0.739	0.167	32.1 ± 5.62	31.9 ± 6.28	0.808	0.138	31.8 ± 5.93	32.3 ± 6.09	0.498	0.179
Morning	10 (8.9)	20 (10.6)	0.387	-	11 (8.5)	19 (11.2)	0.709	-	20 (12.0)	10 (7.5)	0.237	-
Moderate	55 (49.1)	77 (41.0)			57 (43.8)	75 (44.1)			67 (40.4)	65 (48.5)		
Evening	47 (42.0)	91 (48.4)			62 (47.7)	76 (44.7)			79 (47.6)	59 (44.0)		
**Some circadian and chrononutrition parameters**												
Breakfast frequency (Meals/day)	5.4 ± 2.19	5.4 ± 2.19	0.922	0.625	5.3 ± 2.29	5.5 ± 2.11	0.636	0.245	5.6 ± 2.06	5.2 ± 2.33	0.180	0.098
Meals frequency (Meals/day)	4.1 ± 0.93	4.0 ± 0.93	0.595	0.071	4.0 ± 0.90	4.1 ± 0.95	0.308	0.071	4.0 ± 0.98	4.1 ± 0.87	0.479	0.884
< 4	27 (24.1)	55 (29.3)	0.333	-	41 (31.5)	41 (24.1)	0.153	-	48 (28.9)	34 (25.4)	0.494	-
≥4	85 (75.9)	133 (70.7)			89 (68.5)	129 (75.9)			118 (71.1)	100 (74.6)		
After dinner snack (frequency/week)	4.7 ± 2.26	4.6 ± 2.43	0.770	0.405	4.4 ± 2.50	4.8 ± 2.25	0.092	0.069	4.6 ± 2.33	4.7 ± 2.41	0.568	0.617
**Largest meal**												
Breakfast	30 (26.8)	53 (28.1)	0.550	-	40 (30.8)	43 (25.3)	0.342	-	47 (28.3)	36 (26.9)	0.653	-
Lunch	15 (13.4)	33 (17.6)			23 (17.7)	25 (14.7)			29 (17.5)	19 (14.2)		
Dinner	67 (59.8)	102 (54.3)			67 (51.5)	102 (60.0)			90 (54.2)	79 (26.3)		
**Weekdays**												
Sleep duration (h)	8.3 ± 1.23	8.0 ± 1.31	0.077	0.444	8.0 ± 1.29	8.2 ± 1.28	0.126	0.066	8.1 ± 1.41	8.1 ± 1.12	0.946	0.553
Wake up time (h:min)	07:49 ± 0:55	07:41 ± 0:47	0.199	0.956	7:41 ± 0:49	7:46 ± 0:51	0.373	0.204	07:47 ± 0:51	07:40 ± 0:49	0.250	0.147
Sleep onset time (h:min)	23:37 ± 1:09	23:42 ± 1:08	0.486	0.361	23:45 ± 1:08	23:37 ± 1:09	0.347	0.298	23:44 ± 1:11	23:36 ± 1:05	0.373	0.613
**Food timing**												
First eating time (h:min)	09:15 ± 1:48	09:21 ± 1:40	0.638	0.061	9:25 ± 2:32	9:14 ± 1:43	0.326	0.253	9:14 ± 1:40	9:25 ± 1:46	0.368	0.889
Lunch time (h:min)	13:15 ± 1:07	13:19 ± 1:05	0.515	0.355	13:16 ± 1:08	13:18 ± 1:06	0.744	0.760	13:23 ± 1:03	13:08 ± 1:08	0.092	0.106
Dinner time (h:min)	18:48 ± 0:48	18:58 ± 0:54	0.113	0.056	18:54 ± 0:53	18:54 ± 0:51	0.998	0.146	18:57 ± 0:53	18:51 ± 0:51	0.396	0.088
Last eating time (h:min)	21:33 ± 1:36	21:41 ± 1:29	0.461	0.389	21:34 ± 1:29	21:41 ± 1:33	0.530	0.933	21:41 ± 1:35	21:35 ± 1:28	0.595	0.970
Morning latency (min)	78.2 ± 71.68	91.3 ± 80.56	0.147	0.177	82.7 ± 7.34	72.8 ± 5.58	0.074	0.115	79.2 ± 71.52	95.2 ± 83.76	0.083	0.095
Evening latency (min)	123.8 ± 89.58	120.1 ± 81.44	0.717	0.949	128.4 ± 88.05	116.2 ± 81.44	0.221	0.266	121.3 ± 81.22	121.7 ± 88.58	0.966	0.996
Eating midpoint	15:24 ± 1:19	15:31 ± 1:13	0.444	0.538	15:30 ± 1:14	15:27 ± 1:16	0.771	0.869	15:27 ± 1:15	15:30 ± 1:16	0.765	0.724
Eating window (h)	12.3 ± 2.14	12.3 ± 2.01	0.876	0.302	12.1 ± 2.02	12.5 ± 2.08	0.197	0.342	12.4 ± 2.05	12.2 ± 2.05	0.249	0.881
**Weekends**												
Sleep duration (h)	9.8 ± 1.59	9.5 ± 1.32	0.201	0.330	9.6 ± 1.45	9.5 ± 1.42	0.782	0.911	9.5 ± 1.46	9.8 ± 1.38	0.079	0.082
Wake up time (h:min)	10:16 ± 1:37	10:02 ± 1:23	0.197	0.548	10:04 ± 1:25	10:10 ± 1:31	0.555	0.907	10:02 ± 1:25	10:14 ± 1:33	0.246	0.147
Sleep time (h:min)	23:31 ± 1:23	23:32 ± 1:15	0.975	0.694	00:26 ± 1:16	00:34 ± 1:20	0.337	0.998	00:33 ± 1:21	00:28 ± 1:15	0.543	0.821
**Food timing**												
First eating time (h:min)	11:13 ± 1:35	11:12 ± 1:21	0.910	0.281	11:09 ± 1:22	11:15 ± 1:30	0.551	0.402	11:05 ± 1:24	11:21 ± 1:29	0.111	0.058
Lunch time (h:min)	14:00 ± 1:06	14:22 ± 1:18	**0.041**	**0.017**	14:13 ± 1:19	14:13 ± 1:13	0.950	0.861	14:27 ± 1:15	13:56 ± 1:10	**0.006**	**0.004**
Dinner time (h:min)	18:56 ± 0:53	18:52 ± 1:01	0.517	0.463	18:51 ± 1:02	18:56 ± 0:54	0.438	0.271	18:55 ± 1:00	18:52 ± 0:56	0.615	0.218
Last eating time (h:min)	22:06 ± 1:49	22:07 ± 1:42	0.986	0.877	21:55 ± 1:41	22:15 ± 1:47	0.099	0.539	22:07 ± 1:45	22:06 ± 1:44	0.964	0.506
Morning latency (min)	57.0 ± 48.35	68.9 ± 53.98	**0.048**	**0.048**	65.9 ± 53.76	63.4 ± 51.10	0.683	0.752	61.1 ± 48.91	68.6 ± 55.91	0.227	0.221
Evening latency (min)	144.4 ± 89.37	143.9 ± 89.15	0.961	0.846	150.4 ± 95.49	139.2 ± 83.81	0.288	0.415	146.3 ± 88.83	141.3 ± 89.64	0.633	0.533
Eating midpoint	16:40 ± 1:23	16:39 ± 1:15	0.962	0.977	16:32 ± 1:13	16:45 ± 1:22	0.152	0.105	16:36 ± 1:16	16:44 ± 1:21	0.395	0.413
Eating window (h)	10.9 ± 1.98	10.9 ± 1.78	0.916	0.318	10.8 ± 1.84	11.0 ± 1.85	0.279	0.220	11.0 ± 1.89	10.8 ± 1.80	0.191	0.368
Eating jetlag (minutes)	79.3 ± 67.79	72.8 ± 54.16	0.543	0.573	67.7 ± 56.64	81.0 ± 61.28	**0.026**	**0.034**	73.2 ± 56.64	76.1 ± 60.16	0.670	0.592

p^a^ values were unadjusted (Independent t-test was used to analyse differences between minor allele carrier and non-carrier groups). p^b^ values were adjusted for age, gender, and BMI z-scores. The association of the CLOCK gene variants with some circadian and chrononutrition parameters was determined by linear regression. Values are mean ± standard deviation (SD). Pearson's x^2^ test was used for categorical variables. The statistically significant p values are shown in bold (p < 0.05). PSQI, Pittsburgh Sleep Quality Index.

We conducted separate analyses for each sex to evaluate the effects of CLOCK gene polymorphisms on circadian parameters and chrononutrition variables. No significant sex-specific differences were observed (data not shown). However, girls carrying the rs3749474 risk allele had lower sleep durations (*p* = 0.033, Supplementary Figure 9).

### 3.3 Dietary intake

In Table 4, the daily energy and macronutrient intakes of adolescents according to the CLOCK genotypes are presented. Individuals with the CLOCK rs4580704 minor G allele had higher average intakes of total energy, carbohydrates (CHO), protein, total fat, saturated fatty acids (SFA), monounsaturated fatty acids (MUFA), along with polyunsaturated fatty acids (PUFA) compared to those with the CC genotype (*p* < 0.05). However, after adjusting for covariates in the model, only the difference in total energy intake remained statistically significant (*p* = 0.007). Similarly, adolescents with the AA genotype of the CLOCK rs1801260 variant demonstrated higher intakes of dietary energy and fat intake [total, SFA (g), MUFA (g)] compared to carriers of the minor allele. Nonetheless, these associations lost statistical significance after model adjustment.

**Table 4:** Dietary daily energy and macronutrient intake of individuals according to CLOCK genotypes.

	**CLOCK rs3749474**				**CLOCK rs4580704**				**CLOCK rs1801260**			
	**CC (*****n*** = **112)**	**CT**+**TT (*****n*** = **188)**	*p* ^a^	*p* ^b^	**CC (*****n*** = **130)**	**CG**+**GG (*****n*** = **170)**	*p* ^a^	*p* ^b^	**AA (*****n*** = **166)**	**AG**+**GG (*****n*** = **134)**	*p* ^a^	*p* ^b^
**Total dietary intake**												
Energy (kkal)^β^	1,726.2± 452.41	1,785.6 ± 555.0	0.315	0.461	1,649.0 ± 481.65	1,850.6 ± 530.52	**0**.**001**	**0**.**007**	1,816.2 ± 536.76	1,698.5 ± 490.26	**0**.**049**	0.141
CHO (g)	204.6 ± 64.87	208.0 ± 71.40	0.675	0.592	193.6 ± 64.01	216.7 ± 71.02	**0**.**003**	0.646	212.4 ± 70.56	199.7 ± 66.47	0.112	0.430
CHO (%)	48.3 ± 6.93	47.6 ± 6.31	0.390	0.705	47.8 ± 6.49	47.8 ± 6.61	0.972	0.776	47.8 ± 6.27	47.9 ± 6.90	0.920	0.529
Fiber (g)	17.8 ± 6.77	18.4 ± 7.60	0.477	0.653	17.4 ± 6.55	18.8 ± 7.78	0.083	0.474	18.5 ± 7.84	17.8 ± 6.56	0.365	0.203
Protein (g)	59.4 ± 18.64	61.6 ± 21.36	0.352	0.673	57.3 ± 19.40	63.4 ± 20.78	**0**.**010**	0.799	62.5 ± 20.74	58.6 ± 19.79	0.095	0.657
Protein (%)	14.0 ± 2.14	14.3 ± 2.64	0.351	0.546	14.2 ± 2.48	14.2 ± 2.47	0.881	0.842	14.2 ± 2.48	14.2 ± 2.46	0.964	0.933
Fat (g)	72.6 ± 21.23	77.0 ± 26.56	0.119	0.722	70.8 ± 24.04	78.9 ± 24.79	**0**.**005**	0.501	77.9 ± 25.67	72.2 ± 23.26	**0**.**042**	0.579
Fat (%)	37.5 ± 6.24	38.1 ± 5.99	0.438	0.852	38.0 ± 6.20	37.9 ± 6.00	0.882	0.764	37.9 ± 6.17	37.8 ± 5.99	0.889	0.591
SFA (g)	25.7 ± 9.05	27.5 ± 11.46	0.128	0.762	25.2 ± 10.47	28.1 ± 10.63	**0**.**017**	0.937	28.0 ± 10.96	25.4 ± 10.1	**0**.**033**	0.345
SFA (%)	13.4 ± 3.23	13.6 ± 3.22	0.623	0.928	13.5 ± 3.36	13.6 ± 3.12	0.900	0.845	13.7 ± 3.36	13.4 ± 3.04	0.388	0.241
MUFA (g)	25.0 ± 7.68	26.5 ± 10.02	0.147	0.959	24.1 ± 8.29	27.3 ± 9.70	**0**.**003**	0.431	26.9 ± 9.87	24.8 ± 8.27	**0**.**044**	0.728
MUFA (%)	13.2 ± 2.81	13.2 ± 2.94	0.883	0.803	13.1 ± 2.91	13.2 ± 2.88	0.748	0.483	13.2 ± 3.01	13.2 ± 2.75	0.911	0.679
PUFA (g)	15.9 ± 6.58	17.0 ± 7.45	0.188	0.727	15.6 ± 6.69	17.3 ± 7.41	**0**.**041**	0.851	17.1 ± 7.53	15.9 ± 6.61	0.175	0.831
PUFA (%)	8.3 ± 2.79	8.5 ± 2.73	0.625	0.693	8.4 ± 2.77	8.4 ± 2.74	0.856	0.821	8.3 ± 2.66	8.5 ± 2.86	0.607	0.730

p^a^ values were unadjusted (Independent t-test was used to analyse differences between minor allele carrier and non-carrier groups). p^b^ values were adjusted for age, gender, BMI z-score, and energy intake. ^β^Adjusting variables not included in the model when examined as dependent variables for energy intake (p^b^ values were adjusted for age, gender, and BMI z-score). The association of the CLOCK gene variants with dietary intake was determined by linear regression. Values are mean ± standard deviation (SD). The statistically significant p values are shown in bold (p < 0.05). SFA, saturated fatty acids; MUFA, monounsaturated fatty acids; PUFA, polyunsaturated fatty acids.

Additionally, no significant genotype-based differences in energy or macronutrient intake were observed for any CLOCK variants (rs3749474, rs4580704, and rs1801260) when analyzed by specific meal times (breakfast, lunch, dinner, and night-time eating) (*p* > 0.05, see Supplementary Table 1).

When we also evaluated the relationship between dietary energy, macronutrient intakes (carbohydrate, protein, and fat), and biochemical parameters according to minor allel carriers and non-carriers for all three gene variants, we did not find any statistical significance (*p* > 0.05, data not shown).

### 3.4 The effect of CLOCK rs3749474, rs4580704, and rs1801260 variants' interactions with chronotype and sleep quality on dietary intake

**Figure 2:**
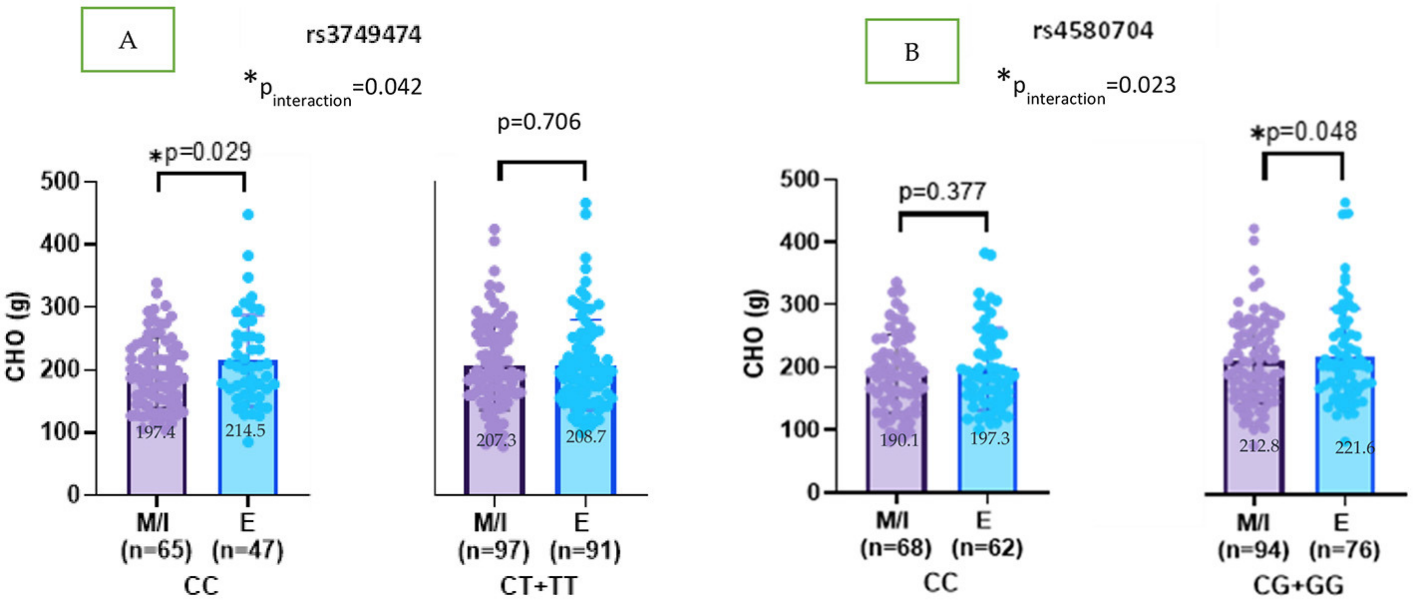
**(A, B)** CLOCK rs3749474 and rs4580704 interaction with chronotype on CHO (g) intakes (adjusted age, gender, BMI z-score and energy intake). Values are mean ± standard deviation (SD). p_interaction_ value was obtained from General Linear model. Other *p* values were obtained from linear regression analysis. M, morning; I, intermediate; E, evening. *p < 0.05.

Supplementary Figures 1–4 illustrate the interaction effects between CLOCK gene variants (rs3749474, rs4580704, and rs1801260) and chronotype on dietary intake patterns. No significant interactions were observed between these CLOCK variants and chronotype in relation to total energy (kcal) and protein (g) intake (see Supplementary Figures 1, 3). For CLOCK rs3749474, a significant interaction with chronotype was found (p_interaction_ = 0.042). Specifically, evening-type individuals carrying the CC genotype consumed more carbohydrates than morning or intermediate chronotype individuals (β = 12.667; *p* = 0.029). In contrast, CHO intake did not differ significantly by chronotype among carriers of the minor T allele (β = −1.354; *p* = 0.706) (Figure 2A).

For CLOCK rs4580704, a similar interaction effect was observed (p_interaction_ = 0.023). Evening chronotype individuals with the G allele consumed significantly more carbohydrates compared to morning/intermediate chronotype individuals (β = 8.999; *p* = 0.048) (Figure 2B).

**Figure 3:**
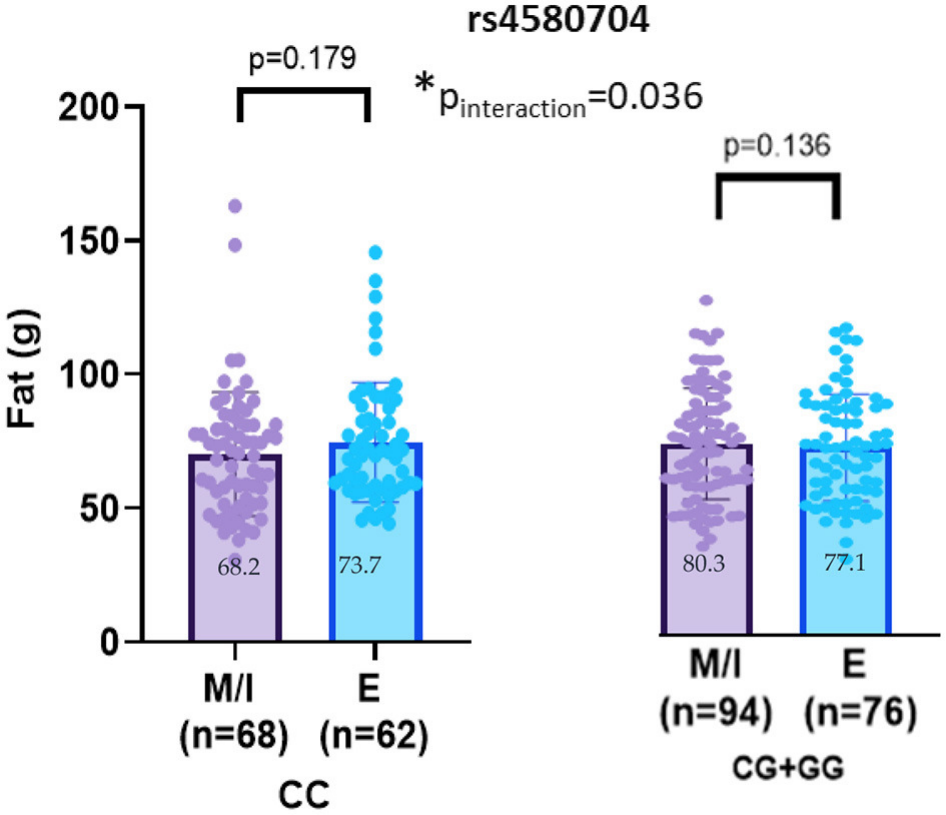
CLOCK rs4580704 interaction with chronotype on fat (g) intakes (adjusted age, gender, BMI z-score and energy intake). Values are mean ± standard deviation (SD). p_interaction_ value was obtained from General Linear model. Other p values were obtained from linear regression analysis (*p* < 0.05). M, morning; I, intermediate; E, evening. *p < 0.05.

No significant interaction effects between chronotype and the CLOCK rs3749474 or rs1801260 variants were found for dietary fat intake (*p* > 0.05). Although an interaction was detected between CLOCK rs4580704 and chronotype for fat consumption (p_interaction_ = 0.036), further analysis by genotypes revealed that this significance disappeared (p > 0.05) (Figure 3).

Furthermore, interactions between sleep quality and CLOCK variants (rs3749474, rs4580704, and rs1801260) did not significantly influence dietary energy, protein, or fat intake (see Supplementary Figures 5–8).

### 3.5 The effect of CLOCK rs3749474, rs4580704, and rs1801260 variants' interaction with chrononutrition parameters, sleep quality, and chronotype on BMI z-score

Table 5 presents the effects of CLOCK rs3749474, rs4580704, and rs1801260 variants and their interactions with chrononutrition parameters, sleep quality, and chronotype on BMI z-score. Key chrononutrition variables, such as eating window, eating midpoint, and morning-evening latency, were calculated as weekly averages based on weekday and weekend data. For individuals carrying the minor allele (CT+TT) of CLOCK rs3749474, a significant positive association was observed between BMI z-score and the frequency of snacking after the last meal (β = 0.134, *p* = 0.003). This suggests that late snacking may contribute to higher BMI z-scores in minor allele carriers.

**Table 5:** The effect of the CLOCK rs3749474, rs4580704, and rs1801260 variants' interaction with chrononutrition parameters, sleep quality, and chronotype on BMI z-score.

	**rs3749474**				**rs4580704**				**rs1801260**			
	**CC**		**CT**+**TT**		**CC**		**CG**+**GG**		**AA**		**AG**+**GG**	
	β **(95CI%)**	***p***	β **(95CI%)**	***p***	β **(95CI%)**	***p***	β **(95CI%)**	***p***	β **(95CI%)**	***p***	β **(95CI%)**	***p***
Eating window	0.118 (–0.146, 0.381)	0.377	0.097 (–0.090, 0.284)	0.307	0.207 (–0.049,0.463)	0.111	0.101 (–0.103, 0.305)	0.330	0.081 (–0.094, 0.256)	0.364	0.192 (–0.109, 0.493)	0.208
Eating jetlag	0.000 (–0.006, 0.005)	0.912	−0.003 (–0.007, 0.001)	0.154	−0.003 (–0.009, 0.002)	0.265	0.000 (–0.005, 0.004)	0.823	0.002 (–0.002, –0.006)	0.364	−0.005 (–0.010, 0.001)	0.084
Eating midpoint	0.657 (–8.956, 10.271)	0.892	−5.240 (–11.580, 1.100)	0.105	0.224 (–9.092, 9.540)	0.962	−5.180 (–12.015, 1.654)	0.136	−8.032 (-14.786, –1.277)	**0.020**	2.480 (–6.589, 11.549)	0.589
Morning latency	0.005 (–0.004, 0.014)	0.265	−0.001 (–0.005, 0.004)	0.819	0.001 (–0.005, 0.007)	0.696	0.003 (–0.004, 0.010)	0.356	0.004 (–0.002, 0.009)	0.207	0.001 (0.006, 0.008)	0.149
Evening latency	0.001 (–0.006, 0.007)	0.845	0.002 (–0.002, 0.006)	0.323	0.004 (–0.001, 0.009)	0.104	0.001 (–0.004, 0.006)	0.740	−0.001 (–0.005, 0.004)	0.794	0.001 (–0.006, 0.008)	0.823
Breakfast frequency/week	−0.116 (–0.287, 0.054)	0.178	−0.099 (–0.202, 0.004)	0.059	−0.059 (–0.189, 0.070)	0.363	−0.178 (–0.311, –0.045)	**0.009**	−0.166 (–0.285, −0.048)	**0.006**	−0.108 (–0.253, 0.037)	0.144
Meal frequency/day	0.202 (–0.159,0.563)	0.270	0.126 (–0.108, 0.360)	0.288	0.240 (–0.082, 0.561)	0.142	0.063 (–0.200, 0.327)	0.635	0.032 (–0.206, 0.270)	0.791	0.367 (0.002, 0.731)	**0.049**
Snacking after the last meal frequency/week	0.023 (–0.136, 0.182)	0.776	0.134 (0.045, 0.223)	**0.003**	0.070 (–0.056, 0.197)	0.273	0.142 (0.031, 0.253)	**0.012**	0.092 (–0.007, 0.191)	0.069	0.084 (–0.045, 0.213)	0.199
Chronotype score	0.010 (–0.049, 0.069)	0.732	−0.004 (–0.042, 0.033)	0.818	−0.009 (–0.062, 0.045)	0.742	0.014 (–0.029, 0.056)	0.527	0.008 (–0.035, 0.050)	0.729	0.000 (–0.052, 0.052)	0.996
PSQI score	−0.021 (–0.109, 0.067)	0.640	0.033 (–0.036, 0.102)	0.347	0.019 (–0.070, 0.109)	0.668	0.010 (–0.061, 0.081)	0.784	0.024 (–0.049, 0.097)	0.518	0.010 (–0.073, 0.093)	0.810

Results of multivariable linear regressions were given. Age, gender, and physical activity level were adjusted. The statistically significant p values are shown in bold (p < 0.05).

Among adolescents with the CLOCK rs4580704 minor G allele, breakfast frequency showed a negative association with BMI z-score (β = −0.178, *p* = 0.009), indicating that higher breakfast frequency may be associated with a lower BMI z-score. Additionally, a positive relationship was noted between BMI z-score and the frequency of snacking after the last meal (β = 0.142, *p* = 0.012).

For individuals with the common AA genotype of CLOCK rs1801260, BMI z-score was negatively associated with both the timing of the eating midpoint (β = −8.032, *p* = 0.020) and breakfast frequency (β = −0.166, *p* = 0.006), suggesting that an earlier eating midpoint and more frequent breakfast consumption are associated with lower BMI z-scores in AA genotype carriers. Conversely, among minor allele carriers of this variant, a positive association was observed between BMI z-score and total meal frequency (β = 0.367, *p* = 0.049), indicating that higher meal frequency may correlate with an elevated BMI z-score in these individuals.

Furthermore, no significant interactions were found between CLOCK gene variants (rs3749474, rs4580704, and rs1801260) and total dietary energy or macronutrient intake in relation to BMI z-score (data not shown).

### 3.6 The effect of CLOCK rs3749474, rs4580704, and rs1801260 variants' interactions with meal energy intake on overweight/obesity risk and evening chronotype

Table 6 summarizes the association between breakfast energy intake, CLOCK gene variants, and the risk of overweight or obesity and evening chronotype among adolescents. When comparing adolescents with high energy intake at breakfast (≥21.0%) to those with the common homozygous CC genotype of the CLOCK rs3749474 gene, the risk of being overweight or obese is three times higher for individuals with low energy intake at breakfast (< 21.0%); the CLOCK rs4580704 gene minor G allele carriers exhibit a twofold higher risk, and individuals with the CLOCK rs1801260 gene AA genotype was approximately 2.5 times higher. No significant differences were observed in CLOCK gene variants and energy distribution in other meals (e.g., lunch, dinner) in relation to the risk of being overweight or obese (Table 6).

**Table 6:** The relationship between energy distribution (%) in meals and the risk of being overweight/obese and evening chronotype according to the CLOCK genotypes.

	**Overweight/obesity**											
	**rs3749474**				**rs4580704**				**rs1801260**			
	**CC**		**CT**+**TT**		**CC**		**CG**+**GG**		**AA**		**AG**+**GG**	
	**OR (95% CI)**	*p* ^a^	**OR (95% CI)**	*p* ^a^	**OR (95% CI)**	*p* ^a^	**OR (95% CI)**	*p* ^a^	**OR (95% CI)**	*p* ^a^	**OR (95% CI)**	*p* ^a^
Breakfast energy (%) < 21.0	3.171 (1.238, 8.120)	**0.016**	1.259 (0.614, 2.580)	0.529	1.731 (0.722, 4.152)	0.219	2.148 (1.043, 4.426)	**0.038**	2.467 (1.143, 5.324)	**0.021**	1.662 (0.702, 3.934)	0.248
Lunch energy (%) ≤ 20.0	1.107 (0.421, 2.908)	0.837	1.229 (0.593, 2.548)	0.580	0.977 (0.414, 2.305)	0.958	1.483 (0.691, 3.183)	0.312	1.627 (0.740, 3.577)	0.226	1.124 (0.465, 2.716)	0.796
Dinner energy (%) ≥34.5	0.578 (0.226, 1.479)	0.253	1.293 (0.614, 2.723)	0.499	1.242 (0.532, 2.895)	0.616	0.683 (0.322, 1.449)	0.320	1.203 (0.532, 2.716)	0.657	0.591 (0.259, 1.351)	0.591
Night energy (%) ≥9.0	0.934 (0.374, 2.333)	0.883	0.754 (0.372, 1.529)	0.434	0.484 (0.209, 1.124)	0.091	0.984 (0.488, 1.984)	0.964	0.793 (0.372, 1.688)	0.547	0.781 (0.358, 1.703)	0.535
	**Evening chronotype**											
	**rs3749474**				**rs4580704**				**rs1801260**			
	**CC**		**CT**+**TT**		**CC**		**CG**+**GG**		**AA**		**AG**+**GG**	
	**OR (95% CI)**	**p** ^b^	**OR (95% CI)**	**p** ^b^	**OR (95% CI)**	**p** ^b^	**OR (95% CI)**	**p** ^b^	**OR (95% CI)**	**p** ^b^	**OR (95% CI)**	**p** ^b^
Breakfast energy (%) < 21.0	1.151 (0.475, 2.789)	0.756	2.001 (1.033, 3.874)	**0.040**	2.054 (0.896, 4.711)	0.089	1.202 (0.616, 2.346)	0.591	1.445 (0.731, 2.859)	0.290	1.861 (0.798, 4.343)	0.151
Lunch energy (%) ≤ 20.0	0.696 (0.273, 1.775)	0.448	0.750 (0.386, 1.456)	0.395	0.556 (0.241, 1.284)	0.169	0.877 (0.434, 1.771)	0.714	0.957 (0.479, 1.910)	0.900	0.543 (0.224, 1.314)	0.543
Dinner energy (%) ≥34.5	2.623 (1.080, 6.374)	**0.033**	0.785 (0.398, 1.548)	0.484	1.286 (0.558, 2.962)	0.555	1.168 (0.585, 2.333)	0.659	1.213 (0.590, 2.497)	0.599	1.365 (0.606, 3.074)	0.452
Night energy (%) ≥9.0	1.272 (0.536, 3.020)	0.585	1.318 (0.687, 2.527)	0.406	2.085 (0.915, 4.751)	0.080	1.044 (0.544, 2.004)	0.898	1.456 (0.733, 2.895)	0.284	1.322 (0.616, 2.833)	0.474

Normal BMI group and morning/intermediate chronotype are considered as reference. Breakfast energy (%) ≥21.0 (reference category = 1), lunch energy (%) >20.0 (reference category = 1), dinner energy (%) < 34.5 (reference category = 1), night energy (%) < 9.0 (reference category = 1). Binary Logistic Regression Analysis was used. p^a^ values were adjusted for age, gender and physical activity level. p^b^ values were adjusted for age, gender, BMI z-scores, and physical activity level. The statistically significant p values are shown in bold (p < 0.05).

Adolescents carrying the rs3749474 minor allele (CT+TT) and consuming < 21.0% of daily energy at breakfast were twice as likely to have an evening chronotype compared to those with higher breakfast energy intake. Individuals with the rs3749474 CC genotype who consumed a high proportion of their daily energy intake in the evening were ~2.5 times more likely to have an evening chronotype (p < 0.05) (Table 6).

An analysis of the interaction between CLOCK gene variants (rs3749474, rs4580704, and rs1801260) and meal-specific energy intake percentages on HOMA-IR risk (defined as a median value of 2.28 or above) revealed no significant associations (data not shown).

## 4 Discussion

This study represents the first comprehensive evaluation of the relationship between CLOCK rs3749474, rs4580704, and rs1801260 polymorphisms in addition to circadian rhythm, metabolic parameters, chrononutrition, dietary intake, as well as dietary intake at meals in adolescents.

Carriers of the minor T allele in the CLOCK rs3749474 variant showed a 2.134-fold increased risk of being overweight or obese. In contrast, no significant differences were observed in anthropometric measurements or the risk of overweight and obesity between minor allele carriers and non-carriers for the CLOCK rs4580704 and rs1801260 variants. However, individuals with the homozygous AA genotype of CLOCK rs1801260 had significantly higher physical activity levels compared to minor allele carriers.

For the CLOCK rs4580704 variant, carriers of the minor G allele had significantly increased dietary energy intake and were more likely to experience eating jetlag. Individuals with the CLOCK rs3749474 minor T allele had later lunch time and longer morning latency on weekends compared to those without the allele. Conversely, those carrying the CLOCK rs1801260 minor allele ate lunch earlier. Additionally, snacking after the last meal was positively correlated with BMI z-scores in minor allele carriers of both the rs3749474 and rs4580704 variants. Minor G allele carriers of rs4580704 demonstrated a negative association between breakfast frequency and BMI z-scores, while those with the rs1801260 minor G allele showed a positive correlation between meal frequency and BMI z-scores. Overall, both minor allele carriers and non-carriers of the CLOCK gene variants exhibited similar biochemical parameters and dietary intake patterns across meals.

### 4.1 Anthropometric measurements, physical activity levels, and biochemical parameters

Although there are studies investigating the link between obesity and different SNPs in the CLOCK gene in various populations, findings regarding their relationship with anthropometric measurements, especially in the child and adolescent population, remain insufficient (29, 31, 32, 61). In these studies conducted on adults, BMI and waist circumference values of individuals with the CC genotype of the CLOCK rs3749474 gene were found to be lower than those with the TT genotype (29), and minor allele carriers at least one copy of the T allele at the CLOCK rs3749474 displayed a significantly higher degree of obesity (weight and BMI) and abdominal obesity (waist circumference) than major allele carriers (62). There are also studies showing no significant difference in anthropometric measurements such as BMI, fat mass, and waist circumference based on the genotype of the CLOCK rs3749474 gene (38, 72). In contrast to prior investigations, a cross-sectional study involving 1268 prepubertal children (aged 6–8 years) in Spain revealed that females carrying the minor allele T of CLOCK rs3749474 exhibited significantly lower BMI compared to non-carriers; no differences were observed in men (73). In this study, in line with findings from prior research (39, 74), adolescents carrying the minor T allele of the CLOCK rs3749474 gene exhibited statistically higher BMI z-scores, body fat (%), and trunk fat (%) compared to those with the homozygous common CC genotype, after adjusting for sex and age (Table 2). Furthermore, our research revealed that those who possess the minor T allele of the CLOCK rs3749474 gene have a two-fold higher risk of becoming overweight or obese than the CC genotype. Age-related factors could influence the association between this gene and anthropometric variables. Studies have indicated that aging can lead to alterations in rhythmic circadian expression, resulting in either a gain or loss of rhythmicity. These differences could help elucidate the inconsistencies in research findings based on age groups (73).

For the other CLOCK gene variants investigated in the current study, CLOCK rs4580704 and rs1801260, no significant differences were found in anthropometric measurements (such as BMI z-score, waist circumference, body fat, and trunk fat) or the risk of overweight or obese between minor allele carriers and non-carriers. For the CLOCK rs4580704 gene, a study conducted in children (aged 6–8 years) in Spain (73) and the studies conducted in adults (72, 74) found similar body weight, BMI, waist circumference, hip circumference, and waist/hip ratio regarding the CLOCK rs4580704 gene. There are studies on the CLOCK rs1801260 gene, one of the most frequently studied CLOCK SNPs in relation to anthropometric variables, have been conducted on adult individuals (38, 75), university students (76), prepubertal children (73), and school-age children (40, 77) and they revealed that the differences in BMI, waist circumference, fat mass, and body weight were not statistically significant according to the genotype. The findings support previous literature regarding anthropometric measurements (38, 40, 75–77). Because CLOCK gene polymorphisms are associated with response to weight loss treatment (41, 74) and there are no studies on the effect of this gene on weight loss or maintenance of healthy body weight in children and adolescents, it is thought that the current study data will contribute to the evaluation of the impact of these CLOCK genotypes on the response to weight management treatment in dietary interventions focused on adolescents.

The circadian rhythm plays a crucial role in maintaining lipid and glucose balance, which is associated with obesity (30). Circadian-clock genes are crucial in regulating food intake by controlling both central circadian mechanisms and peripheral clock-controlled genes. This regulation leads to variations in plasma and tissue phospholipid levels by managing transporters and proteins involved in phospholipid metabolism. Recently, these genes have gained significant attention as potential pharmacological targets. Targeting circadian-clock genes may offer therapeutic benefits for enhancing resistance to insulin resistance, diabetes, obesity, metabolic syndrome, and atherosclerosis (78). In adults with coronary heart disease, blood lipid levels were evaluated according to the genotypes of the CLOCK rs3749474, rs4580704, and rs1801260 genes. The results showed that for all three genes, lipid levels (including total cholesterol, LDL, HDL, triglycerides, Lp(a), ApoB, ApoA1, and hsCRP) were similar between those carrying the risk alleles and those who did not. Additionally, after following either a low-fat diet or a Mediterranean diet for 12 months, it was found that individuals with the CC major allele showed a greater reduction in high-sensitivity C-reactive protein and a significant increase in the HDL/apolipoprotein A1 ratio compared to those carrying the GG or CG minor alleles. No significant gene-diet interaction was observed for the other gene polymorphisms (79). In a study of 1,100 participants from the Genetics of Lipid Lowering Drugs and Diet Network, no significant difference was found between CLOCK rs3749474, rs4580704 and rs1801260 genes and blood lipids, fasting glucose, insulin and HOMA-IR values (72). In this study, serum lipid levels, fasting blood glucose, and insulin resistance were similar between minor allele carriers and non-carriers of the CLOCK gene variants (rs3749474, rs4580704, and 1801206) CLOCK rs4580704 gene minor allele carriers (CG+GG) exhibited higher AST levels than non-carriers (*p* = 0.014). However, this difference disappeared after adjusting for age, gender, and BMI z-score. Similar findings to our study have been reported across diverse populations (39, 73, 75, 79, 80), and similar results for adolescents have been proven in this study. Based on the available data, there is no evidence to suggest that polymorphisms in the CLOCK gene variants (rs3749474, rs4580704, and rs1801260) pose a risk to blood lipid levels, insulin, HOMA-IR levels, or liver enzymes in Turkish adolescents. We also evaluated the relationship between dietary energy and macronutrient intakes (carbohydrate, protein, and fat), and biochemical parameters according to minor carriers and non-carriers for all three gene variants, we did not find any statistical significance. Circadian-clock genes regulate key metabolic pathways, but it's not only the CLOCK gene that plays a role. Other key genes in the circadian machinery, such as BMAL1, PER, CRY, and REV-ERB, are also crucial in regulating metabolic processes and have been shown to interact with pathological states like insulin resistance, diabetes, lipid metabolism, and obesity (81). Although our study did not reveal a significant influence of CLOCK gene polymorphisms on pathological parameters, existing evidence supports the idea that other circadian rhythm-related genes may play a more critical role in regulating metabolic functions and pathological outcomes. Further research targeting the broader circadian molecular machinery, including genes like BMAL1, PER, CRY, and REV-ERB, may provide deeper insights into how circadian regulation affects metabolic diseases such as insulin resistance, diabetes, and obesity.

Physical activity is one of the most commonly assessed environmental factors in gene-environment studies on obesity (82). Previous studies have found no significant difference in physical activity levels according to CLOCK rs3749474, rs4580704, and rs1801260 genotypes (38, 75, 83). Although previous studies found no significant differences in physical activity levels for various CLOCK gene variants, including rs3749474, rs4580704, and rs1801260 (36, 73, 81), a study on Japanese university students showed lower hourly physical activity levels in TC+CC carriers (minor allele carriers) than in TT carriers (common homozygotes) for rs1801260 on Saturdays, even when adjusting for sex and BMI (76). Additionally, Bandin et al. found that women with moderate obese who carry the CLOCK rs1801260 minor allele (C) exhibited a delayed acrophase, characteristic of “evening-type” individuals. These women were also less active than TT carriers, began their activities later in the morning, and experienced greater daytime sleepiness (84). Although our study found no significant differences in circadian parameters, the results were consistent with previous studies regarding physical activity levels (76, 84). We observed that adolescents with the rs1801260 homozygous AA genotype have significantly higher physical activity levels (PAL: 1.6 ± 0.29) compared to minor G allele carriers (PAL: 1.4 ± 0.26; *p* = 0.023) which may indicate a potential protective effect against future obesity, highlighting the importance of lifestyle factors in modifying genetic susceptibility to obesity. However, our cross-sectional study cannot establish a direct causal relationship between physical activity and protection from obesity for this or other genotypes. Prospective, longitudinal studies would be necessary to confirm whether the increased physical activity observed in AA genotype carriers is indeed protective, emphasizing the need for further research to clarify these relationships.

### 4.2 Circadian and chrononutrition parameters

CLOCK gene constitutes a strong candidate gene group for examining the underlying genetic predisposition in determining chronotype. This gene controls circadian rhythms and plays an important role in regulating energy homeostasis (85, 86). During adolescence, there is a significant tendency for sleep patterns to shift toward later in the evening, peaking toward the later stages of adolescence (87). This shift is influenced by the interaction of genetic, environmental, and social factors with the adolescent development process (88). In this study, no significant difference was found in both chronotype total scores and chronotype distributions between individuals carrying and not carrying minor alleles in the CLOCK gene (rs3749474, rs4580704, rs1081260) (Table 4). Research on adult people has revealed that carriers of the rs1801260 C minor allele exhibited an evening preference compared to individuals with the TT genotype (42, 80, 89, 90). However, other studies found no association (91–93). The EPIC study revealed that the chronotype score and distribution of chronotypes did not differ according to genotypes of CLOCK rs3749474, rs4580704, and rs1801260. These findings corroborate our research (8). On the other hand, late chronotype is associated with a decrease in insulin sensitivity and energy expenditure in addition to an increase in glucose, insulin, and triglyceride levels, independent of obesity. The present study included the influence of the genetic factor. No significant relationship was found when examining the interaction between CLOCK gene variants and chronotype score on BMI z-score (Table 5). The results of this study revealed that a minority of the participants (10% of the entire sample) were morning chronotypes, while a significant majority (46%) were evening chronotypes. This may prevent the effect of the chronotype score on BMI z-score from being seen. Thus, future studies need to support these data by ensuring the homogeneity of the chronotype distribution.

Genetic factors can cause sleep disorders (94). The CLOCK gene is the most important gene associated with the endogenous molecular circadian clock. It encodes proteins that control circadian rhythm. Mutations in the circadian CLOCK gene affect sleep quality by causing changes in the circadian rhythm cycle (94–96). Some circadian gene polymorphisms, including rs1801260, rs11932595, and rs6843722, have been associated with sleep parameters in studies (40, 89, 94). In a study conducted on surgical nurses with and without sleep disorders, PSQI total scores and subcomponents (excluding hypnotic drugs) of individuals with the CLOCK rs1801260 gene TT genotype were higher than in individuals with the CC genotype. Also, it was determined that the CLOCK rs1801260 gene TC genotype was protective compared to the TT genotype for sleep disorder (OR = 0:434, 95% CI: 0.240–0.785) in multiple logistic regression analysis (94). In the EPIC-Spain study, no variation was found in sleep quality concerning genetic variants of CLOCK rs3749474, rs4580704, and rs1801260 (8). Consistent with previous study findings, our study aligns with these findings; no significant association was found in the sleep quality of adolescents according to the CLOCK rs3749474, rs4580704, and rs1801260. The results of this study indicate that in Turkish adolescents, the CLOCK gene variants (rs3749474, rs4580704, and rs1801260) represent no candidate gene group relevant to sleep quality. Adolescents are a group that may be more vulnerable to disturbances in their sleep-wake cycle due to several causes that cause them to go to bed later (such as social media and screen usage) and have inconsistent sleep patterns (such as sleeping in on weekends and taking daytime naps) (97, 98). These factors may have obscured the significance of genetic differences.

This is the first study to evaluate chrononutrition parameters according to CLOCK 3749474, rs4580704, and rs1801260 genotypes along with their effects on BMI z-score. According to CLOCK variants, one of the most important findings regarding chrononutrition features according to CLOCK variants is that CLOCK rs4580704 gene G minor allele carriers had more eating jetlag duration compared to the CC genotype. Research examining eating jetlag based on genotype was found in the literature (99). Barragán et al. found no significant association between eating jetlag and CLOCK rs4580704, FTO-rs9939609, ve TAS2R38-rs713598 gene polymorphisms (99). Eating jetlag (the difference in eating time between workdays and free days) was associated with higher BMI, markers of adiposity, cardiovascular events, and reduced glycemic control (58, 100). In our previous study on adult individuals, eating jetlag was not associated with overweight/obesity and sleep quality (56). This study also examined the effect of genetic factors on adolescents. However, the interaction of CLOCK gene variants and eating jetlag did not effect on BMI z-scores (Table 5). This most likely results from the idea that the timing of the first and last eating occasion may be affected by waking time, which alters more on weekdays than weekends due to work schedules along with social restraints. Additionally, this study was conducted cross-sectionally. Therefore, in future studies to be conducted prospectively in different groups, there is a need to evaluate the effect of interaction of CLOCK variants' interaction with eating jetlag on obesity and metabolic parameters and to understand the underlying mechanisms.

In this study, meal timings were similar between individuals who were carriers and non-carriers of the minor allele of the CLOCK gene (rs3749474, rs4580704, and rs180126) on weekdays. Yang et al. found that the meal times for lunch and dinner were similar for individuals with the CLOCK rs1801260 gene minor risk allele carriers or noncarriers, which supports our findings on weekends (80). Similar to this study, a prior study in the general population revealed no association between meal times (breakfast, lunch, and dinner times) according to the CLOCK rs4580704 genotypes (99). Individuals with the minor allele carrier of the CLOCK rs3749474 gene tended to have a later lunch meal, while CLOCK rs1801260 gene minor allele carriers tended to have an earlier lunch meal on weekends. Contrary to our findings, some research has indicated that adults with overweight or obesity who carry the minor allele of the CLOCK rs1801260 gene ate breakfast later (42, 80). According to the CLOCK rs1801260 genotype, the results are inconsistent regarding lunch and dinner times (42, 80). The discrepancy in various results may be attributed to variations in the number of subjects and study groups, lifestyle and environmental factors, as well as in meal timings and definitions resulting from cultural eating patterns.

### 4.3 The effect of (rs3549474, rs4580704, and rs1801260) interaction of CLOCK gene variants with chronotype and sleep quality on dietary energy and macronutrient intake

Genetic variations may be the root cause of the different responses to dietary treatments (43). This study demonstrated that adolescents with the minor T allele carriers of the CLOCK rs374947 gene consumed similar amounts of dietary energy, CHO, protein, fat, and fiber intake compared to the CC genotype. These findings align with previous research (38, 68). Adolescents who carry the CLOCK rs4580704 gene minor allele eat more total energy, CHO, protein, and fat [total, SFA, MUFA (g) as well as PUFA (g)] compared to individuals who carry the CC genotype. However, after the covariant variables were included in the model, statistical significance was only observed in energy intake (*p* = 0.007). Our study confirms previous studies in line with existing literature (41, 101). Unlike our study, the dietary energy intake of individuals with the CC genotype, was statistically higher than that of individuals carrying the risk allele (GG+CG) (68). Most research investigating the association between dietary energy intake and the CLOCK rs1801260 gene (based on genotype or minor allele carrying status) did not find statistical significance (38, 41, 68).

However, a study conducted on adults with overweight or obesity in Iran revealed that individuals with the common TT genotype had lower energy consumption compared to those with the CC and CT genotypes (42). In this study, when covariate variables were included in the analysis, CLOCK rs1801260 gene minor allele carriers and noncarriers showed similar dietary energy and macronutrient intakes, consistent with the findings of most studies in the literature (38, 41, 68). At the same time, it was determined that the interaction of SNPs in the CLOCK rs3749474/rs4580704/rs1801260 gene with dietary energy and macronutrient consumption had no influence on BMI z-score (data not shown). Adolescence is a period in which the tendency toward unhealthy eating habits is generally high (eating behaviors deteriorate, skipping meals, consumption of fast food, and sugary drinks become more common). It is seen that these habits are similar in most individuals. Therefore, the reason why dietary intakes according to CLOCK genotype and diet-gene interaction affect BMI z-score in this study may be due to this similarity.

Sleep patterns and circadian rhythm change during adolescence. However, to our knowledge, no study has been found in the literature on whether the interaction of sleep quality, chronotype, and the CLOCK gene polymorphisms affects dietary intake. This study revealed that individuals with the CLOCK rs3749474 CC genotype and G minor allele carriers of rs4580704 with an evening chronotype consumed significantly more CHO than morning individuals. As in the Iranian study (42), no significant difference was observed for the CLOCK rs1801260 gene in this study. Previous studies have found higher CHO intake in individuals with late chronotypes, regardless of gene (102, 103). In the current study, it was determined that this situation differs according to CLOCK genotypes. Since circadian clock genes are regulators of circadian rhythm that modulate the expression of Peroxisome Proliferator-Activated Receptor (PPAR), which is a family of transcription factors involved in cellular lipid metabolism (lipolysis and lipogenesis) (35), high-fat diets can disrupt the circadian modulation of metabolic factor expression in peripheral tissues, causing obesity. Low-CHO and high-fat ketogenic diets have been found to affect peripheral circadian clocks and tissue-specific oscillation of clock control genes (104). Such changes in diet composition are associated with abnormal daily changes in the hormones leptin, glucose, and insulin (41). However, appetite hormones were not evaluated in this study. It would be beneficial to confirm these results by including appetite hormones in a larger sample of different ethnic and age groups in the future.

The significance of the circadian rhythm in governing human eating behavior and metabolism has been recognized for some time. Nevertheless, while the distribution of energy intake throughout the day and its potential link with obesity remain unclear, a positive association was observed between evening energy intake and obesity (105). Only one study in the literature examined the distribution of dietary intakes at meals according to the genotypes of the CLOCK rs3749474 gene, and found that both the total energy and macronutrient intakes (%) and the distribution rates of these nutrients in breakfast, lunch, dinner, and snacks were similar according to genotypes (39). In this study, dietary energy intake at meals for the CLOCK rs3749474 gene supports the previous study (39). Furthermore, this study demonstrated for the first time that genetic variants of the CLOCK rs4580704 and rs1801260 exhibit similarity regarding meal distribution (Supplementary Table 1). On the other hand, the logistic regression analysis revealed that low energy intake (%) at breakfast increased the risk of overweight/obesity in common homozygous genotypes for the CLOCK rs3749474 and rs1801260 (OR: 3.171; OR: 2.467, respectively), as well as minor G allele carriers for the CLOCK rs4580704 (OR: 2.148). It was determined that the energy distribution in other meals had no effect on the risk of being overweight/obese according to CLOCK genotypes.

In addition, the current study is the first to evaluate the effect of energy distribution at meals on overweight or obesity in adolescents according to CLOCK rs3749474, rs4580704, and rs1801260 genotypes. Previous studies have found that genotypic differences in the CLOCK gene are essential in dietary intervention responses to body weight loss or metabolic parameters (41, 106). Garcia-Rios et al. found a positive link between change in dietary fat intake and change in BMI in adult women with CLOCK rs3749474 gene T allele carriers following a 12-week dietary intervention (106). Carriers of the minor G allele of CLOCK rs1801260 were more resistant to weight loss and metabolic changes in response to an energy-restricted than the AA homozygous (74). To our knowledge, no research has been conducted on the impact of CLOCK genotypes, meal-specific energy distribution, and their interactions with other chrononutrition characteristics on chronotype and obesity parameters. Genetic variations in circadian clock genes are linked to changes in circadian rhythm (chronodisruption), which may indicate a reduced response to therapy (107). One of the most significant challenges in weight-loss treatments is detecting good predictors of success. The analysis of circadian genes, dietary intake, chronotype, and chrononutrition behaviors (snacking, breakfast frequency, eating window, etc.) could be used to increase the success of such treatments.

Chronotype significantly influences nutritional intake throughout the day (56, 108). Most evening chronotype, energy, and macronutrient intakes occurred at dinner time or night (56, 103, 108), while energy intake was lower at breakfast (103). In this study, individuals carrying the minor allele of the CLOCK rs3749474 gene were twice as likely to have an evening chronotype compared with individuals with a high breakfast energy intake. Individuals with the CLOCK rs3749474 gene CC genotype who consume high energy intake at dinner are almost 2.5 times more likely to exhibit an evening chronotype (*p* < 0.05). The results of the present study highlight CLOCK rs3749474 genotype-specific differences in the association between meal energy intake (especially breakfast and dinner energy %) and chronotype.

### 4.4 The effect of (rs3549474, rs4580704 and rs1801260) interaction of CLOCK gene variants with chrononutrition parameters, sleep quality and chronotype on BMI z-scores

Chrononutrition has many components, such as meal regularity, meal frequency, meal time, skipping breakfast, the largest meal, dinner, evening delay, night eating, eating duration/window, and food consumption at meals (22–24). Later meal times, higher calorie intake in the evening, and diurnal variability in the daily energy intake rhythm could lead to metabolic dysfunction through circadian misalignment (100, 109–111). This study detected a positive relationship between BMI z-score and frequency of snacking after the last meal in minor allele carriers of both CLOCK rs3749474 and rs4580704 gene variants. Furthermore, individuals with the homozygous AA genotype of rs1801260 along with the minor G allele carriers of CLOCK rs4580704 revealed a significant negative link between breakfast frequency and BMI z-score. Breakfast consumption is an important indicator of overall health. This meal provides the body's energy and nutrient requirements for the body. It is frequently regarded as one of the most essential meals of the day due to its ability to activate the metabolism (112). When breakfast is skipped, meal portions are generally larger, and the frequency of snack consumption is higher during the rest of the day. Therefore, eating breakfast is a preventive strategy for regulating energy balance, reducing body fat, and reducing the risk of type 2 diabetes as well as metabolic syndrome (113). Encouraging adolescents to snack less at night may lead to an increase in their consumption of breakfast meals. If adolescents prefer to consume snacks at night, it is essential to replace unhealthy snacks such as chocolate, biscuits, chips, and sugary drinks with healthy choices such as vegetables, fruits, milk, and yogurt, both for adequate and balanced nutrition and for gaining correct eating habits. In this study, genotypic differences in CLOCK genes were important in the effect of breakfast and snacking after the last meal consumption on BMI z-scores.

Holmbäck et al. found that obesity was lower in adults who consumed six or more meals a day than in those who consumed three or fewer meals a day (114). According to the UK National Health and Nutrition Survey, children had a greater risk of developing abdominal obesity, particularly those who consumed snacks frequently and had more frequent meals. However, this association was found to be inconsistent among adolescents. It has been stated that this is due to the difference in the definitions of meals and snacks (115). This study found that among people who carry the CLOCK rs1801260 gene G minor allele, meal frequency was positively related to their BMI z-score. No study has comprehensively investigated the relationship between CLOCK gene variants and chrononutrition behaviors. Recent discussions in nutrition treatment of obesity have highlighted the significance of eating time and time-restricted nutrition. Therefore, in future studies, in addition to the importance of circadian CLOCK genotypes in individualized nutrition in weight loss programs, the role of chrononutrition parameters such as meal frequency, frequency of eating breakfast, and frequency of consuming snacks in this relationship should be considered.

It is important to keep in mind that this study has several limitations. Firstly, as the current study was conducted cross-sectionally, it is not possible to establish a causal relationship. However, these findings could serve as the basis for future prospective studies to assess and confirm the actual causal relationship. Secondly, although validated questionnaires were used to collect information on sleep patterns and chronobiological preferences, the use of self-reported data presents the possibility of recall bias and misrepresentation, which might affect the accuracy of the findings. In future studies, evaluating sleep quality using more objective methods, such as polysomnography, would be beneficial. Thirdly, participants may need to accurately recall or misreport their food intake, leading to underestimation or overestimation of actual consumption. The tendency of individuals with overweight or obese to underreport their dietary intake could introduce bias and lead to inconsistent findings. Also, taking 24-h dietary intake for 2 days may not reflect long-term dietary habits or trends, limiting the ability to accurately assess overall dietary patterns along with their interaction with circadian rhythms and gene polymorphisms accurately. Fourth, the direct functional effects of CLOCK SNPs on CLOCK gene expression and circadian regulation have not been evaluated. Therefore, investigating gene expression levels is valuable in interpreting some mechanisms and represents an important area for future research. Fifth, this study is the relatively small and homogeneous sample size, which may restrict the generalizability of our findings. Expanding research with larger cohorts and a more ethnically diverse sample is essential for gaining comprehensive insights into the genetic diversity of the circadian system and its implications for chrononutrition, dietary intake, and chronobiological phenomena.

## 5 Conclusion

In summary, this study revealed that CLOCK rs3749474 gene minor T allele carriers have higher anthropometric measurements (BMI z-score, body fat, trunk fat) and the risk of being overweight/obese. The CLOCK rs4580704 gene minor G allele carriers exhibit statistically higher dietary energy intake and eating jetlag than the CC genotype after adjustment for confounders. It was found that the interaction of CLOCK gene variants with chronotype, sleep quality, eating window/jetlag, and morning-evening latency had no significant effect on BMI z-score. The frequency of snacking after the last meal was positively correlated with BMI z-score in minor allele carriers of the CLOCK rs3749474 and rs4580704. The minor G allele carriers of CLOCK rs4580704 revealed a negative link between breakfast frequency and BMI z-score. In addition, individuals with the rs1801260 minor G allele showed a positive connection between their BMI z-score and meal frequency. In CLOCK gene variants, minor allele carriers and non-carriers had similar biochemical parameters (*p* > 0.05).

The impact of chrononutrition parameters (breakfast, meal frequency, and snacking after the last meal) on BMI z-score is partially modulated by the variability in the CLOCK gene variants. Our findings suggest that modifying dietary recommendations based on genotypes of individuals can enhance the effectiveness of dietary treatments and lead to better health outcomes. Further research is required to examine additional CLOCK SNPs and other circadian genes in adolescents to understand the relationship between the circadian system and the development of obesity. Additionally, future studies should enhance the efficacy of weight loss programs by refining individualized approaches that consider CLOCK genotype, chronotype, chrononutrition behaviors, in addition to the timing and distribution of food intake at meals.

It is necessary to conduct additional research to determine the connection between sex hormones, pubertal development, and CLOCK gene polymorphisms in order to identify any potential obesogenic pathways causing circadian rhythm disruption.

## Data Availability

The datasets presented in this study can be found in online repositories. The names of the repository/repositories and accession number(s) can be found below: https://www.ncbi.nlm.nih.gov/snp/rs3749474; https://www.ncbi.nlm.nih.gov/snp/rs4580704; https://www.ncbi.nlm.nih.gov/snp/rs1801260.
