# Supplementary material for: Association between CLOCK gene polymorphisms with circadian rhythm, chrononutrition, dietary intake, and metabolic parameters in adolescents
Source: Front Public Health. 2024 Dec 18;12:1435460. doi: 10.3389/fpubh.2024.1435460 (PMC11689662; doi:10.3389/fpubh.2024.1435460)
Supplement: Supplementary file 1 [file Table_1.docx]

Supplemental Table 1. Distribution of daily dietary intakes of individuals according to the CLOCK genotypes.

|  | CLOCK rs3749474 | | | | CLOCK rs4580704 | | | | CLOCK rs1801260 | | | |
| --- | --- | --- | --- | --- | --- | --- | --- | --- | --- | --- | --- | --- |
|  | CC (n=112) | CT+TT (n=188) | p^1^ | p^2^ | CC (n=130) | CG+GG (n=170) | p^1^ | p^2^ | AA (n=166) | AG+GG (n=134) | p^1^ | p^2^ |
| Distribution of energy (day) ^β^ |  |  |  |  |  |  |  |  |  |  |  |  |
| Breakfast (%) | 21,1 ± 12,27 | 20 ± 12,63 | 0,427 | 0,514 | 20,6 ± 12,58 | 20,2 ± 12,46 | 0,798 | 0,746 | 21,4 ± 12,5 | 19,2 ± 12,42 | 0,136 | 0,146 |
| Lunch (%) | 20,5 ± 14,59 | 20,7 ± 13,23 | 0,915 | 0,963 | 20,8 ± 14,83 | 20,4 ± 12,86 | 0,827 | 0,799 | 20,1 ± 12,74 | 21,2 ± 14,89 | 0,514 | 0,540 |
| Dinner (%) | 34,2 ± 12,87 | 35,2 ± 13,91 | 0,553 | 0,520 | 34,1 ± 13,73 | 35,4 ± 13,36 | 0,416 | 0,321 | 34,4 ± 13,44 | 35,4 ± 13,64 | 0,542 | 0,611 |
| Night (%) | 11,6 ± 9,79 | 10,3 ± 9,76 | 0,302 | 0,412 | 10,4 ± 9,45 | 11,1 ± 10,03 | 0,500 | 0,633 | 10,3 ± 9,66 | 11,4 ± 9,92 | 0,358 | 0,274 |
| Distribution of CHO (TEI%) |  |  |  |  |  |  |  |  |  |  |  |  |
| Breakfast (%) | 18,8 ± 11,82 | 18,2 ± 12,87 | 0,634 | 0,791 | 18,6 ± 13,26 | 18,3 ± 11,87 | 0,864 | 0,914 | 19,4 ± 12,52 | 17,2 ± 12,35 | 0,126 | 0,098 |
| Lunch (%) | 20,6 ± 15,34 | 20,6 ± 13,64 | 0,981 | 0,813 | 20,8 ± 15,34 | 20,4 ± 13,45 | 0,818 | 0,456 | 20,3 ± 13,3 | 21,0 ± 15,44 | 0,684 | 0,524 |
| Dinner (%) | 34,2 ± 12,84 | 34,4 ± 14,51 | 0,877 | 0,709 | 33,5 ± 14,47 | 35,0 ± 13,44 | 0,375 | 0,086 | 34,0 ± 13,5 | 34,8 ± 14,41 | 0,639 | 0,950 |
| Night (%) | 11,6 ± 9,13 | 11,4 ± 10,2 | 0,800 | 0,927 | 11,4 ± 9,97 | 11,5 ± 9,69 | 0,944 | 0,601 | 11,0 ± 9,9 | 12,0 ± 9,67 | 0,356 | 0,214 |
| Distribution of Protein (TEI%) |  |  |  |  |  |  |  |  |  |  |  |  |
| Breakfast (%) | 22,9 ± 14,07 | 20,3 ± 13,77 | 0,125 | 0,206 | 21,3 ± 14,23 | 21,2 ± 13,72 | 0,955 | 0,625 | 22,1 ± 13,89 | 20,3 ± 13,95 | 0,275 | 0,167 |
| Lunch (%) | 21,7 ± 16,3 | 21,7 ± 15,02 | 0,980 | 0,771 | 22,1 ± 16,43 | 21,4 ± 14,76 | 0,722 | 0,304 | 21,6 ± 15,15 | 21,8 ± 15,93 | 0,931 | 0,700 |
| Dinner (%) | 37,7 ± 14,88 | 39,6 ± 14,95 | 0,268 | 0,206 | 38,5 ± 15,11 | 39,3 ± 14,83 | 0,644 | 0,307 | 37,8 ± 15,35 | 40,3 ± 14,32 | 0,132 | 0,230 |
| Night (%) | 9,9 ± 10,49 | 7,8 ± 9,15 | 0,078 | 0,075 | 7,5 ± 8,68 | 9,3 ± 10,38 | 0,094 | 0,302 | 8,0 ± 9,37 | 9,2 ± 10,11 | 0,315 | 0,164 |
| Distribution of Fat (TEI%) |  |  |  |  |  |  |  |  |  |  |  |  |
| Breakfast (%) | 23,4 ± 14,71 | 22,1 ± 14,07 | 0,460 | 0,547 | 22,9 ± 13,66 | 22,3 ± 14,81 | 0,707 | 0,948 | 23,7 ± 14,33 | 21,2 ± 14,2 | 0,135 | 0,115 |
| Lunch (%) | 20,0 ± 15,07 | 20,4 ± 14,18 | 0,799 | 0,925 | 20,4 ± 15,4 | 20,1 ± 13,81 | 0,866 | 0,384 | 19,4 ± 13,39 | 21,3 ± 15,75 | 0,277 | 0,151 |
| Dinner (%) | 34,2 ± 14,14 | 34,8 ± 14,34 | 0,733 | 0,531 | 34,2 ± 15,26 | 34,8 ± 13,47 | 0,721 | 0,217 | 33,7 ± 14,52 | 35,7 ± 13,87 | 0,210 | 0,458 |
| Night (%) | 11,0 ± 10,57 | 9,3 ± 10,26 | 0,170 | 0,170 | 9,1 ± 9,86 | 10,6 ± 10,77 | 0,222 | 0,459 | 9,5 ± 10,04 | 10,5 ± 10,83 | 0,427 | 0,266 |

p^1^ values were unadjusted (Independent t test was used to analyse differences between minor allel carrier and non carrier groups)

p^2^ values were adjusted for age, BMI z score, gender and energy intake.

^β^ Adjusting variables not included in the model when examined as dependent variable for energy intake (p^2^ values were adjusted for age, gender and BMI z score).

Association of the CLOCK gene variants with dietary intake was determined by linear regression.
