# Supplementary material for: Association between CLOCK gene polymorphisms with circadian rhythm, chrononutrition, dietary intake, and metabolic parameters in adolescents
Source: Front Public Health. 2024 Dec 18;12:1435460. doi: 10.3389/fpubh.2024.1435460 (PMC11689662; doi:10.3389/fpubh.2024.1435460)
Supplement: Supplementary file 2 [file Data_Sheet_1.pdf]

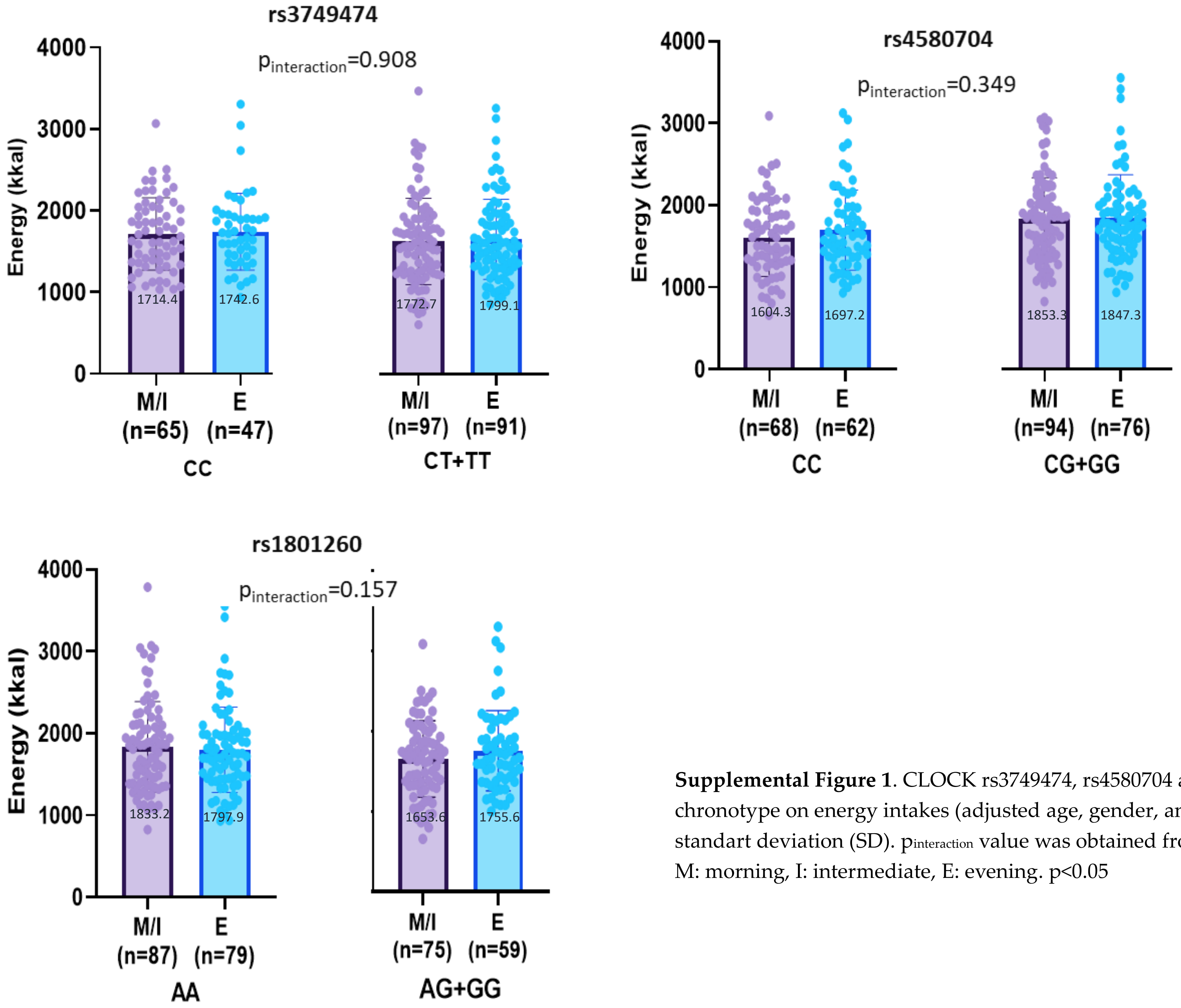

Supplemental Figure 1. CLOCK rs3749474, rs4580704 and rs1801260 SNP interaction with chronotype on energy intakes (adjusted age, gender, and BMI z score). Values are mean ± standart deviation (SD). $p_{interaction}$ value was obtained from General Linear model. Abbrevations: M: morning, I: intermediate, E: evening. $p<0.05$

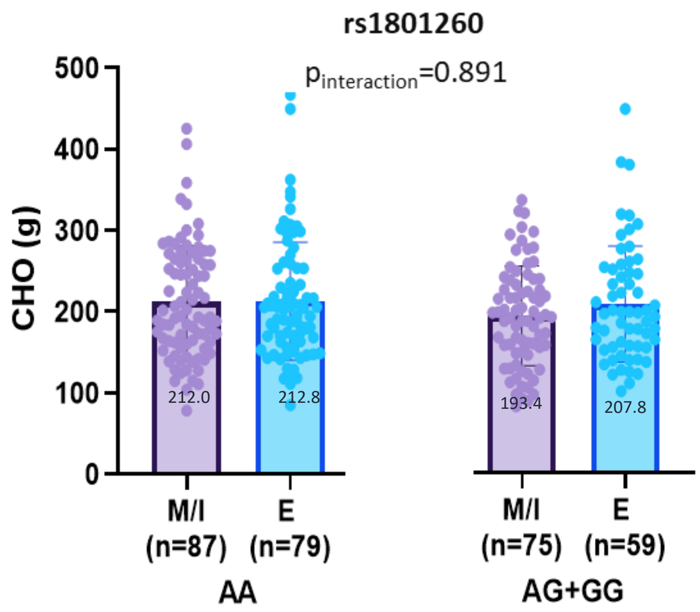

Supplemental Figure 2. CLOCK rs1801260 SNP interaction with chronotype on CHO (g) intakes (adjusted age, gender, BMI z score and energy intake). Values are mean ± standart deviation (SD). $p_{interaction}$ value was obtained from General Linear model. Abbrevations: M: morning, I: intermediate, E: evening. $p<0.05$

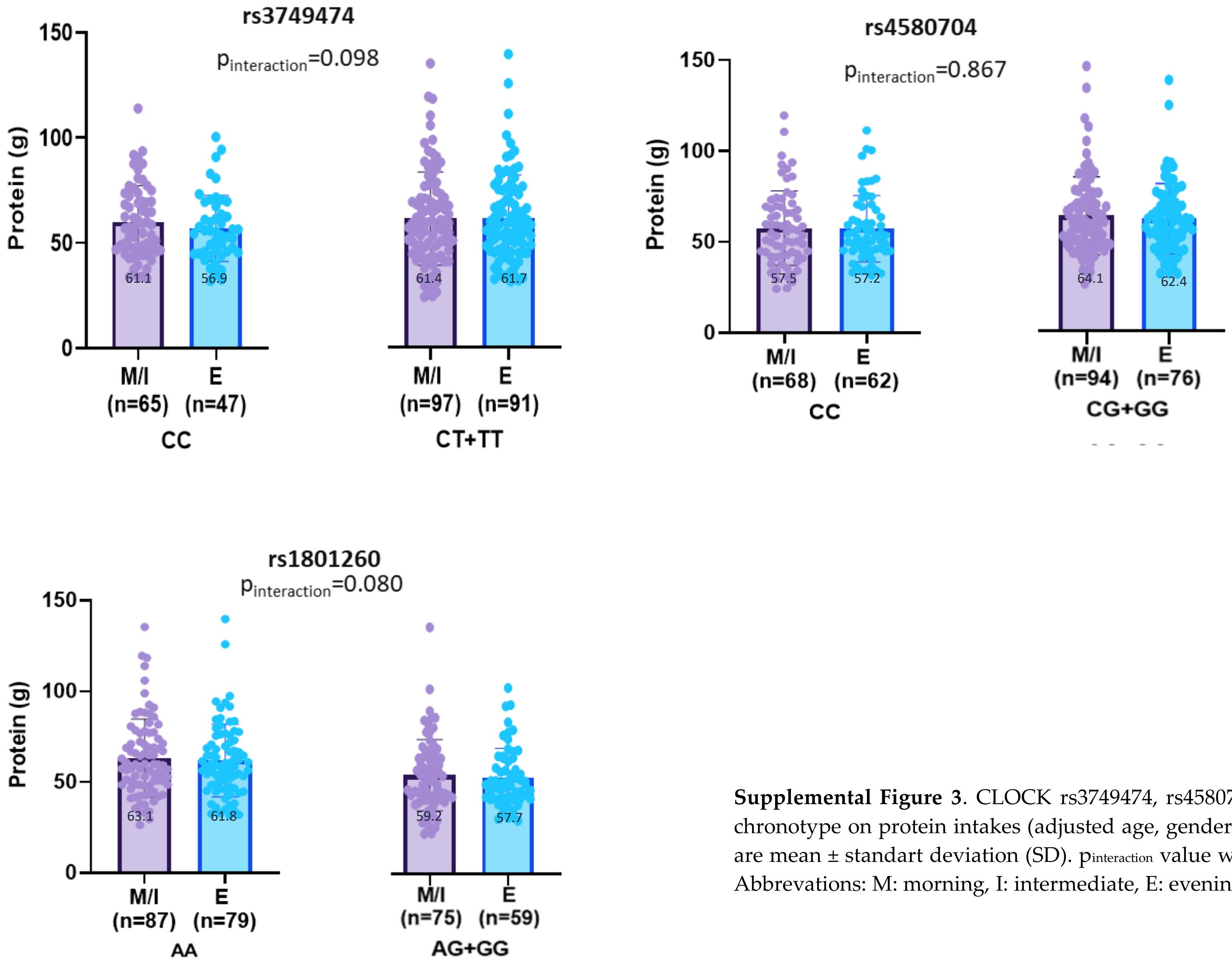

Supplemental Figure 3. CLOCK rs3749474, rs4580704 and rs1801260 SNP interaction with chronotype on protein intakes (adjusted age, gender, BMI z score and energy intake). Values are mean ± standart deviation (SD). $p_{interaction}$ value was obtained from General Linear model. Abbrevations: M: morning, I: intermediate, E: evening. $p<0.05$

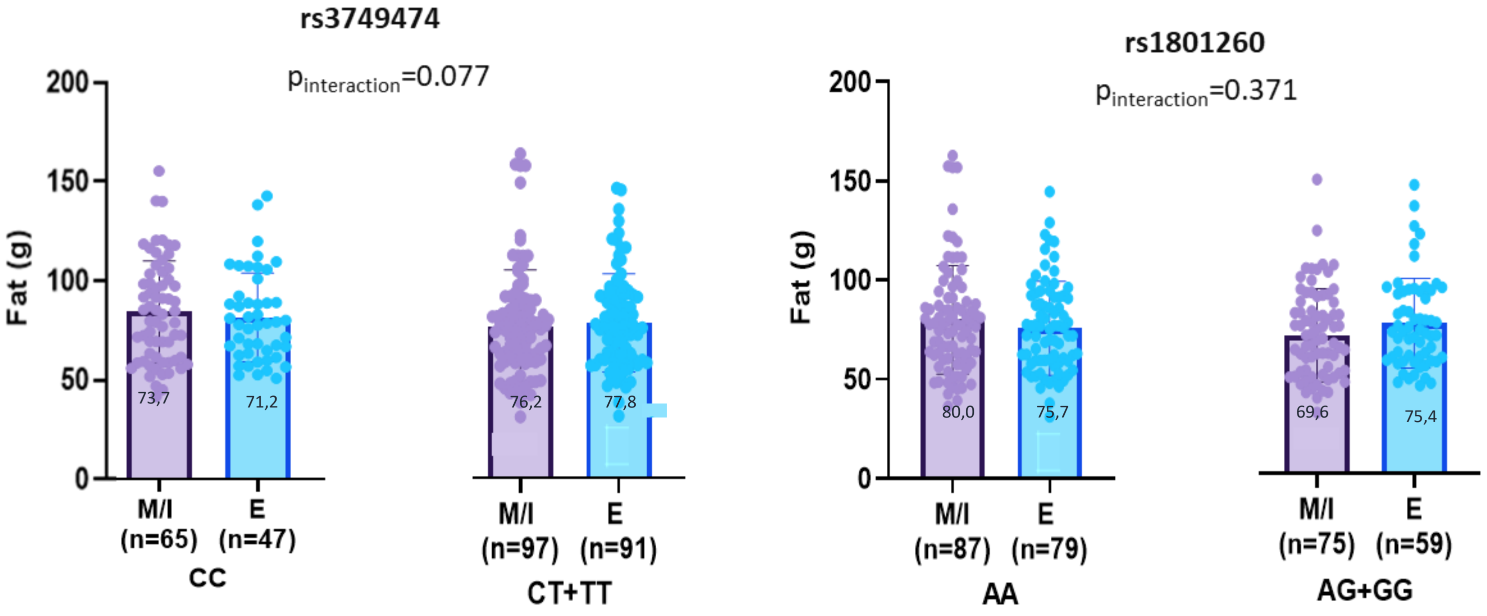

Supplemental Figure 4. CLOCK rs3749474, and rs1801260 SNP interaction with chronotype on fat intakes (adjusted age, gender, BMI z score and energy intake) Values are mean ± standart deviation (SD). $p_{interaction}$ value was obtained from General Linear model. Abbrevations: M: morning, I: intermediate, E: evening. $p<0.05$

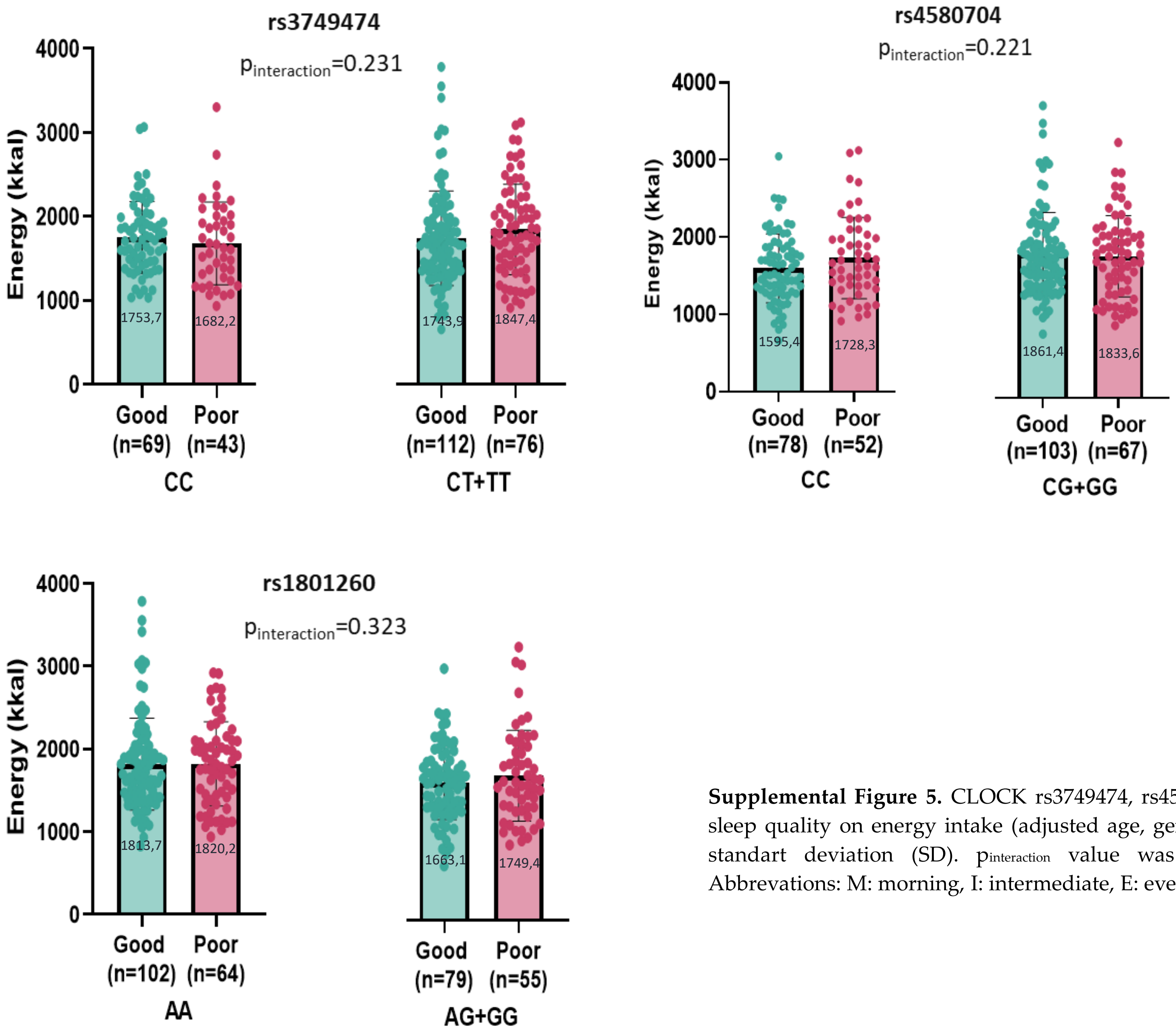

Supplemental Figure 5. CLOCK rs3749474, rs4580704 and rs1801260 SNP interaction with sleep quality on energy intake (adjusted age, gender, and BMI z score). Values are mean ± standart deviation (SD). $p_{interaction}$ value was obtained from General Linear model. Abbrevations: M: morning, I: intermediate, E: evening. $p<0.05$

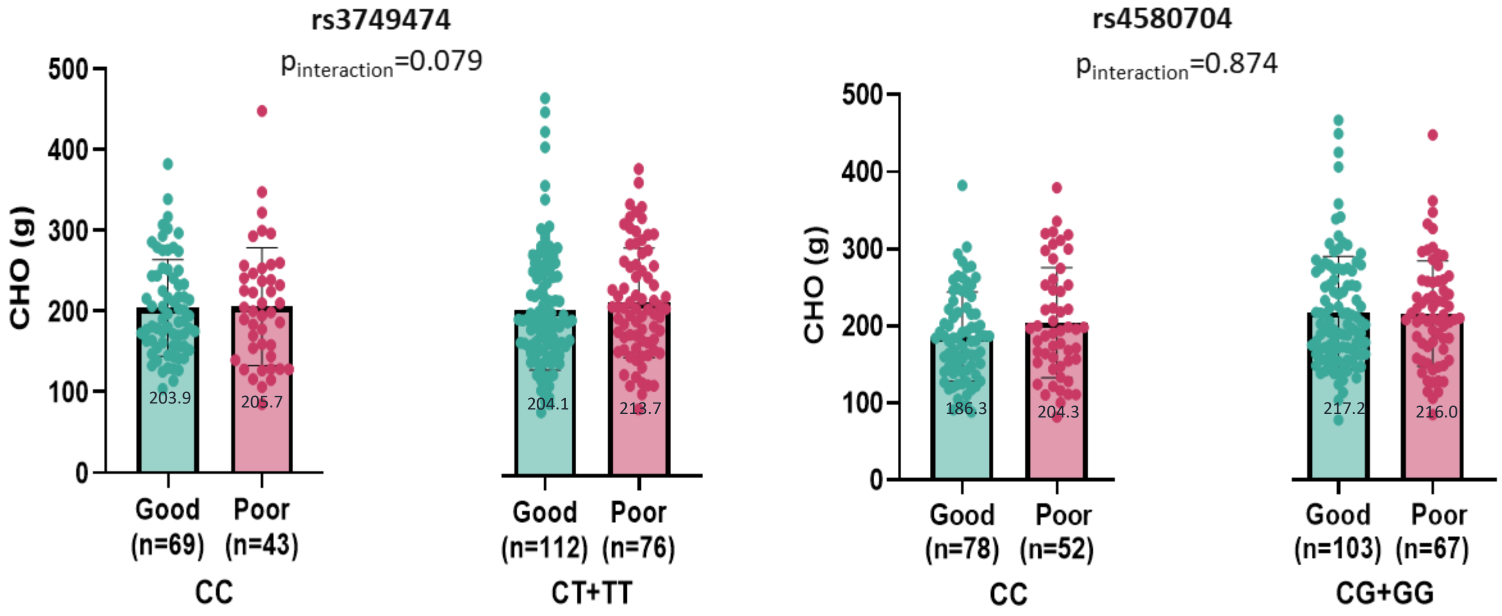

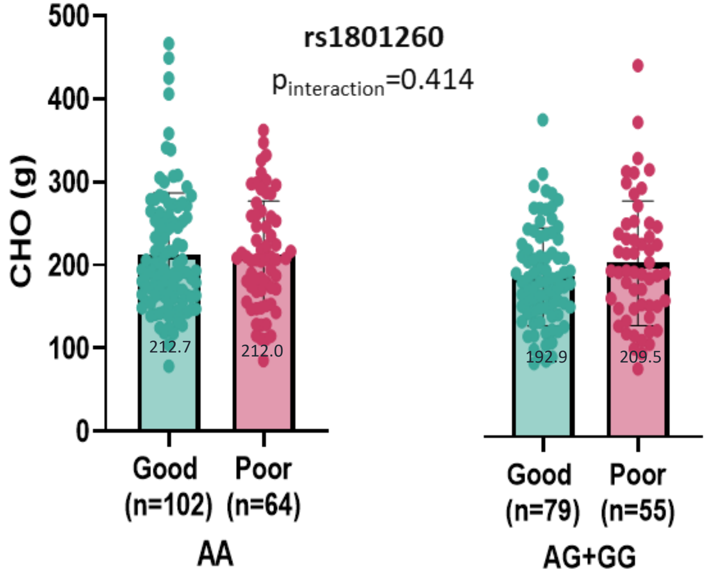

Supplemental Figure 6. CLOCK rs3749474, rs4580704 and rs1801260 SNP interaction with sleep quality on CHO intake (adjusted age, gender, BMI z score and energy intake). Values are mean ± standart deviation (SD). $p_{interaction}$ value was obtained from General Linear model. $p<0.05$

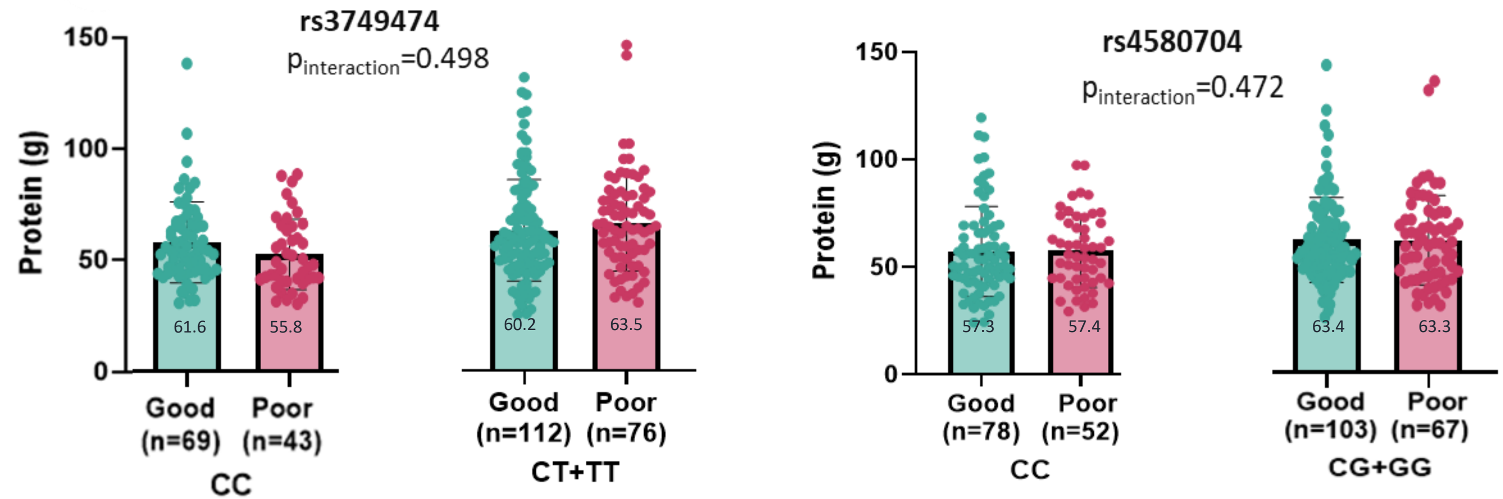

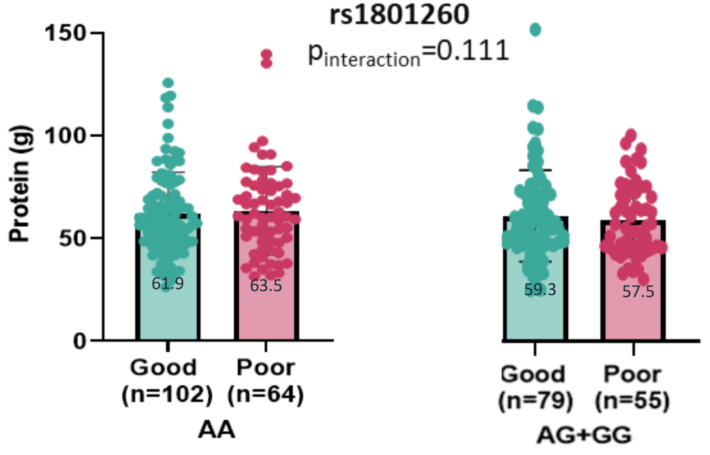

Supplemental Figure 7. CLOCK rs3749474, rs4580704 and rs1801260 SNP interaction with sleep quality on protein intake (adjusted age, gender, BMI z score and energy intake). Values are mean ± standart deviation (SD). $p_{interaction}$ value was obtained from General Linear model. $p<0.05$

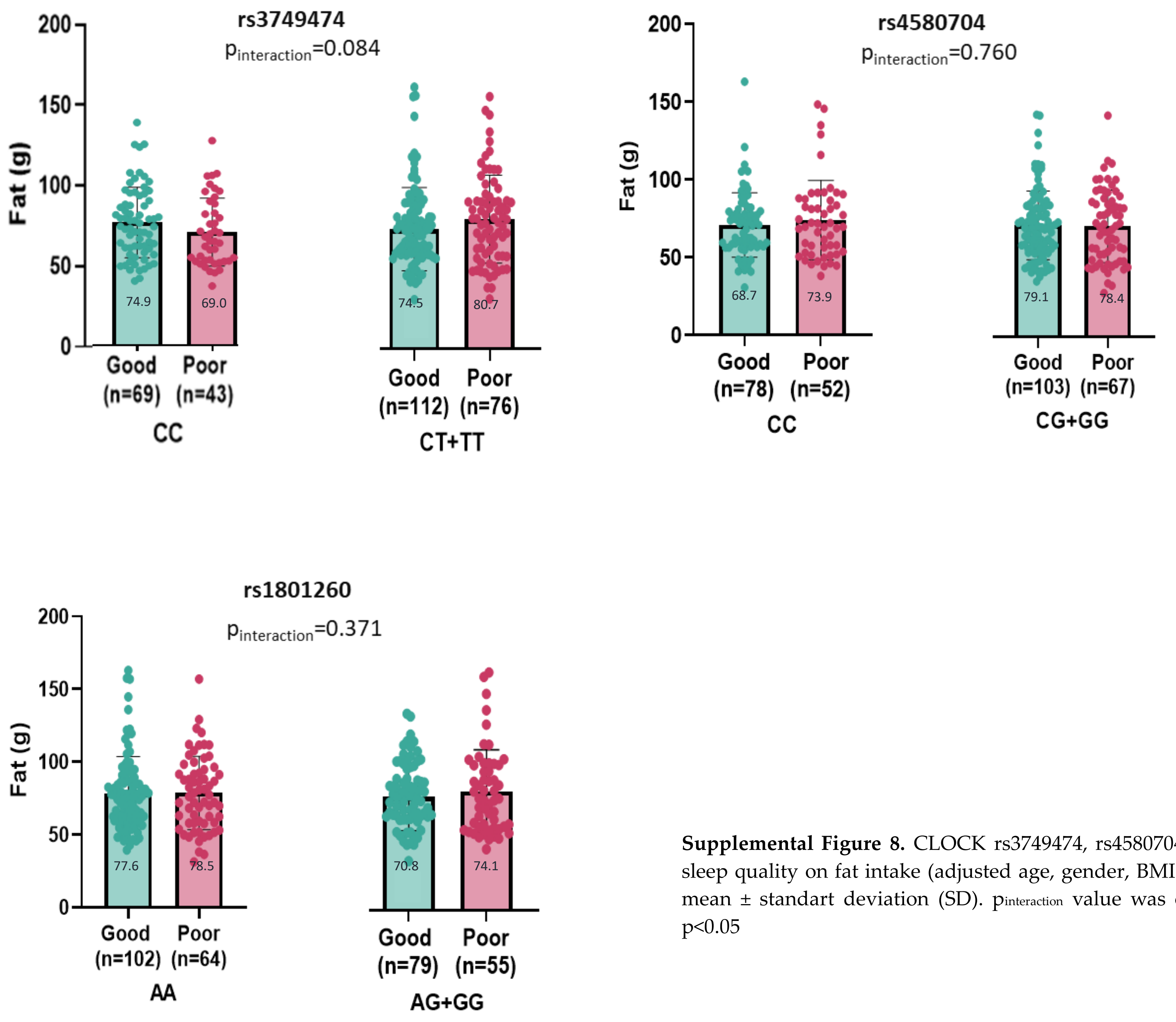

Supplemental Figure 8. CLOCK rs3749474, rs4580704 and rs1801260 SNP interaction with sleep quality on fat intake (adjusted age, gender, BMI z score and energy intake) Values are mean ± standart deviation (SD). $p_{interaction}$ value was obtained from General Linear model. $p<0.05$

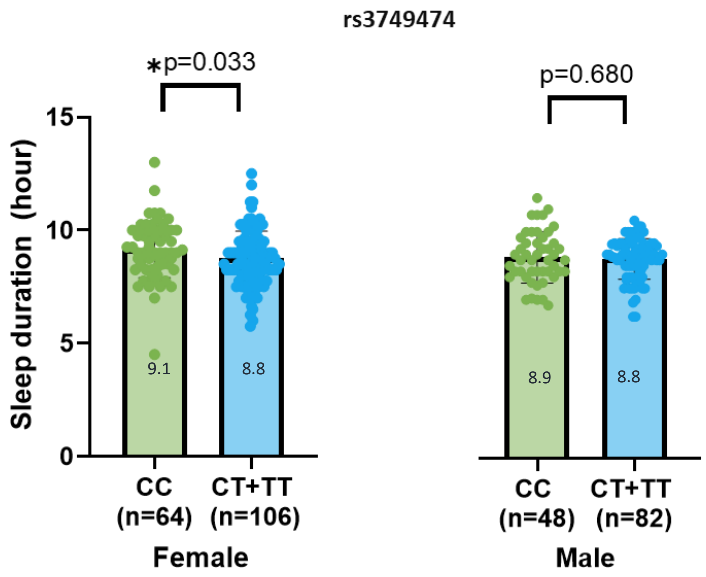

Supplemental Figure 9. Sleep durations of male and female individuals according to their CLOCK rs3749474 genotypes (adjusted age, and BMI z score). Values are mean ± standart deviation (SD). p value was obtained from Linear Regression. $p<0.05$.
